# Molecular design revitalizes the low-cost PTV-polymer for highly efficient organic solar cells

**DOI:** 10.1093/nsr/nwab031

**Published:** 2021-02-12

**Authors:** Junzhen Ren, Pengqing Bi, Jianqi Zhang, Jiao Liu, Jingwen Wang, Ye Xu, Zhixiang Wei, Shaoqing Zhang, Jianhui Hou

**Affiliations:** School of Chemistry and Biological Engineering, University of Science and Technology Beijing, Beijing 100083, China; State Key Laboratory of Polymer Physics and Chemistry, Institute of Chemistry, Chinese Academy of Sciences, Beijing 100190, China; State Key Laboratory of Polymer Physics and Chemistry, Institute of Chemistry, Chinese Academy of Sciences, Beijing 100190, China; CAS Key Laboratory of Nanosystem and Hierarchical Fabrication, CAS Center for Excellence in Nanoscience, National Center for Nanoscience and Technology, Beijing 100190, China; School of Chemistry and Biological Engineering, University of Science and Technology Beijing, Beijing 100083, China; State Key Laboratory of Polymer Physics and Chemistry, Institute of Chemistry, Chinese Academy of Sciences, Beijing 100190, China; State Key Laboratory of Polymer Physics and Chemistry, Institute of Chemistry, Chinese Academy of Sciences, Beijing 100190, China; State Key Laboratory of Polymer Physics and Chemistry, Institute of Chemistry, Chinese Academy of Sciences, Beijing 100190, China; CAS Key Laboratory of Nanosystem and Hierarchical Fabrication, CAS Center for Excellence in Nanoscience, National Center for Nanoscience and Technology, Beijing 100190, China; School of Chemistry and Biological Engineering, University of Science and Technology Beijing, Beijing 100083, China; State Key Laboratory of Polymer Physics and Chemistry, Institute of Chemistry, Chinese Academy of Sciences, Beijing 100190, China; School of Chemistry and Biological Engineering, University of Science and Technology Beijing, Beijing 100083, China; State Key Laboratory of Polymer Physics and Chemistry, Institute of Chemistry, Chinese Academy of Sciences, Beijing 100190, China

**Keywords:** organic solar cell, low-cost, simple chemical structure, poly(thienylene vinylene)

## Abstract

Developing photovoltaic materials with simple chemical structures and easy synthesis still remains a major challenge in the industrialization process of organic solar cells (OSCs). Herein, an ester substituted poly(thiophene vinylene) derivative, PTVT-T, was designed and synthesized in very few steps by adopting commercially available raw materials. The ester groups on the thiophene units enable PTVT-T to have a planar and stable conformation. Moreover, PTVT-T presents a wide absorption band and strong aggregation effect in solution, which are the key characteristics needed to realize high performance in non-fullerene-acceptor (NFA)-based OSCs. We then prepared OSCs by blending PTVT-T with three representative fullerene- and NF-based acceptors, PC_71_BM, IT-4F and BTP-eC9. It was found that PTVT-T can work well with all the acceptors, showing great potential to match new emerging NFAs. Particularly, a remarkable power conversion efficiency of 16.20% is achieved in a PTVT-T:BTP-eC9-based device, which is the highest value among the counterparts based on PTV derivatives. This work demonstrates that PTVT-T shows great potential for the future commercialization of OSCs.

## Teaser

An organic solar cell based on a newly designed and low-cost PTV polymer exhibits over 16% efficiency, demonstrating that the conjugated polymers that have faded out from the field can be revitalized by ingenious molecular design.

## INTRODUCTION

Organic solar cells (OSCs) have drawn much attention due to their unique advantages of being lightweight, flexible, having large-area manufacturability through a low-cost solution coating process and so on [1,2]. In very recent years, the power conversion efficiencies (PCEs), particularly in the OSCs based on non-fullerene acceptors (NFAs), were remarkably boosted to over 18% owing to the rapid development of both photovoltaic/interfacial materials and device fabrication techniques [3–6]. Among the OSCs presenting state-of-the-art PCEs, the choices of polymer donors are limited to several systems, such as PBDB-TF (also known as PM6) and its derivatives [7,8], D18 [3], PTQ10 [9], PTzBI-*d*F [10] and PBTATBT-4f [11] (Fig. S1), which benefit from both desirable opto-electrical properties and, more importantly, fine optimization of the blend morphology when these donor polymers work together with the most popular NFAs, i.e. ITIC [12,13], IT-4F [14] and Y6 [15,16] systems. However, these donor materials were designed with complex molecular structures, by constructing the conjugated backbones with fused heterocyclic systems or introducing halogen atoms, to achieve suitable light absorption spectra and matched energy levels with NFAs, resulting in critical cost issues and scale-up difficulties, which have been deemed as a great obstacle for the future commercialization of OSCs.

In order to find polymer donors with low synthetic cost, two main factors should be taken into consideration. Firstly, functional groups that can only be introduced into the polymers by tedious synthetic methods should be avoided. For example, to reduce synthetic cost of PBDB-TF, a polymer with high-cost fluorine substituted groups, we designed a chlorinated polymer PBDB-TCl [8], which demonstrated a PCE of 14.4%. However, although the synthetic method of PBDB-TCl is much easier than PBDB-TF, the overall synthetic approach of the polymer is still too long to get a low-cost material. Secondly, chemical structures of the polymers should be relatively simple. In past years, noticeable efforts have been devoted to the simple conjugated polymer donors with high photovoltaic performance. For instance, Wang *et al.* synthesized TQ1, a polymer based on thiophene and 2,3-bis-(3-octyloxyphenyl)quinoxaline, and demonstrated a PCE of 6% [17]; Li *et al.* designed a polymer based on thiophene and fluorine-substituted quinoxaline, namely PTQ10, and realized a PCE of 12.70% [9], which was further improved to 16.21% by utilizing Y6 as the acceptor [18]. So far, although a few advances have been made, designing simple polymer donors with high photovoltaic performance and good adaptability to different acceptors is still a great challenge.

In the early stage of OSCs research, polythiophenes (PTs) [19–22], especially poly(3-hexylthiophene) (P3HT), played a critical role as electron donor in OSCs when fullerenes were predominantly adopted as acceptor in a device. Nevertheless, they have been faded out in the highly efficient NFA-based OSCs, because it was quite difficult to form the favorable nanoscale phase separation between PTs and NFAs [19], and only very few PTs that exhibited a pre-aggregation effect in organic solvents could demonstrate over 10% PCEs (shown in Fig. S2) [20–22]. Poly(thienylene vinylene)s (PTVs), a type of low-cost polymers with simple chemical structures, had caught research interest because of their red-shifted absorption spectra and good hole mobilities compared to PTs [23,24], and many PTVs were designed and applied in OSCs [25–28]. However, as shown in Fig. S2, PTVs showed inferior photovoltaic properties in OSCs due to unknown reasons, no matter when they were blended with fullerene or non-fullerene acceptors [25,29]. Even though the photovoltaic performance of PTVs and PTs is not as good as the highly efficient polymers, those classic conductive polymers still have potential to achieve high PCEs and low-cost features simultaneously due to some favorable intrinsic characteristics, for instance, tunable band gaps, matched energy levels with NFAs and high carrier mobilities.

**Figure 1.**
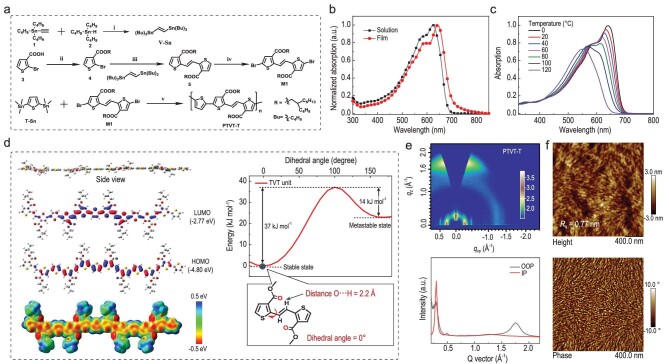
(a) The synthetic routes of PTVT-T. (b) Normalized UV-vis absorption spectra of PTVT-T in chloroform and in solid film. (c) TD-Abs of PTVT-T from 0°C to 120°C with a 20°C interval in chlorobenzene. (d) Theoretical calculation results of the conjugated segments: the side view, LUMO and HOMO distributions, the ESP distribution of tetramers of PTVT-T together with the twisting barriers and the distances between O and H atoms of ester functionalized TVT model. (e) The GIWAXS pattern and the 1D cut-line profile and (f) AFM height and phase images of PTVT-T neat film.

In this contribution, we designed and synthesized a new polymer as shown in Fig. 1a, namely poly(bis(2-butyloctyl) [2,2′ : 5′,2″-terthiophene]-4,4″-dicarboxylate-5,5′-diyl-vinylene) (PTVT-T). As the repeating backbone unit in PTVT-T consists of oligothiophene and vinylene, it can be regarded as a derivative of PTV. This new polymer was synthesized via a five-step route, using low-cost commercially available compounds as raw materials. In solution state, PTVT-T exhibits good solubility and strong interchain aggregation effect, which is proven by temperature-dependent light absorption (TD-Abs) characterization. In solid thin film, PTVT-T demonstrates an absorption spectrum similar to PBDB-TF, a broadly used polymer donor for highly efficient OSCs, and the backbone of PTVT-T tends to form a face-on packing mode. PTVT-T can work well with a few representative fullerene- and non-fullerene-based acceptors, such as PC_71_BM, IT-4F and BTP-eC9 (abbreviated to eC9 in this work). In particular, an optimal PCE of 16.20% can be achieved in PTVT-T:eC9-based OSCs, which is much higher than the results obtained from the other PTV or PT derivatives [19–22,29] and also very close to that of state-of-the-art OSCs [30–32].

## RESULTS AND DISCUSSION

Unlike polymers with complex chemical structures, PTVs and PTs have fewer positions that could be modified for tuning their electronic and morphological properties. With regard to electronic properties, PTVs and PTs naturally have relatively broad band gaps and good hole mobilities [19,25], which could match well with the representative low band gap NFAs like IT-4F and eC9, but their highest occupied molecular orbital (HOMO) levels are too high to get satisfactory open circuit voltages (*V*_OC_) in OSCs. In the conjugated polymers for OSC applications, fluorine substitutions were widely used to realize low-lying HOMO levels but led to a significant increase in synthetic costs. In this newly designed polymer, we introduced two ester groups with bulky alkyl chains to simultaneously get a low-lying HOMO level and a planar conjugated backbone. As shown in Fig. 1d for the theoretical calculations, the HOMO and lowest unoccupied molecular orbital (LUMO) levels are −4.80 and −2.77 eV, respectively, and the π-electron orbitals are evenly delocalized on the backbone. For the (*E*)-1,2-di(thiophen-2-yl)ethene (TVT) unit, the distance between the oxygen on the ester group and the hydrogen on the vinylene bond is 2.20 Å, which is slightly shorter than their van der Waals radii (2.48 Å) [33], implying an obvious O···H non-covalent interaction. In addition, the energy barrier from the stable state to the metastable state is 37 kJ mol^−1^, and the barrier is 14 kJ mol^−1^ from the metastable state to the stable state, implying that the TVT unit tends to rotate to its stable state and the dihedral angle between thiophene and ethylene in TVT is about zero (see Fig. 1d). Furthermore, the stable state with lowest energy locates at 0 degree, indicating that the molecule favors a conformation in which the sulfur on the thiophene and the hydrogen on the vinylene stay in the same direction rather than the opposite one. Therefore, a stable planar backbone conformation can be obtained for PTVT-T. In addition, as shown in Fig. 1d, PTVT-T has a negative surface electrostatic potential (ESP) value along the conjugated backbone, indicating that PTVT-T has a typical electron-donating characteristic.

The detailed synthetic method of PTVT-T is provided in the experimental section of the Supporting Information (SI). The V-Sn can be synthesized in hundreds of grams from widely available raw materials, followed by a four-step reaction to get the target polymer PTVT-T. In order to investigate the ‘low-cost’ characteristic of PTVT-T, we compared the chemical structures, synthesis steps, MOC (material-only cost) of PTVT-T and some typical high-performance polymer donors, including PBDB-TF, D18, PTzBI-*d*F, PTQ-10 and PBTATBT-4f [34]. The evaluation criteria and detailed comparative data are shown in Table 1, Fig. S1 and Table S1. It can be found that PTVT-T not only possesses the advantages of fewer synthesis steps (five steps), but also a significantly lower cost of raw materials than that of the other materials, indicating its huge potential in the future commercialization of OSCs.

**Table 1.** The photovoltaic performance, synthesis steps, MOC and price of some typical highly efficient polymer donors with PCE over 16% and PTVT-T in this work.

Polymer	PCE (%)	Steps^a^	MOC^b^ ($/g)	Price^c^ ($/g)	Ref.
PBDB-TF	17.8	12	46.9	3380	[35]
D18	18.2	17	63.4	3850	[3]
PTQ10	16.2	3	149.2	1650	[18]
PTzBI-*d*F	17.3	21	539.8	4200	[10]
PBTATBT-4f	16.1	15	55.3	2470	[11]
PTVT-T	16.2	5	35.0	—	This work

^a^The synthesis routes of the polymers are started with the rule that the raw materials are commercially available in hectograms or kilograms (excluding the catalysts)

^b^MOC: material-only cost, which is the cost of the raw materials used for polymer synthesis. The cheap materials, including organic solvents, inorganic substances (acids, bases and salt), solvents for post-treatment and water, are not included

^c^The prices were quoted from material suppliers and the website, and may be changed with subsequent updates.

By employing a 2-butyloctyl chain on each of the ester groups, PTVT-T can be easily dissolved in common solvents such as chloroform, chlorobenzene and dichlorobenzene. The number-averaged molar mass (Mn) of PTVT-T is 3.7 × 10^4^ g mol^−1^ with a dispersity (*Đ*) of 2.34 and the thermal stability of PTVT-T was investigated by thermo gravimetric analysis (TGA), and as shown in Fig. S3a, the thermal decomposition temperature (*T*_d_) was 360°C.

As shown in Fig. 1b, the solid film of PTVT-T exhibits an absorption band ranging from 400 to 700 nm, with an absorption coefficient of 9.4 × 10^4^ cm^−1^. The optical band gap (*E*_g_^opt^) is 1.76 eV based on the absorption onset at 704 nm. According to recent works [11,18], high-performance polymer donors for NFA-based OSCs usually show a strong aggregation effect in solution, and the aggregation in the processing solution can be maintained into the coated blend film, in which aggregation-induced polymer channels, and nanoscale phase separation morphology with interpenetrating networks, can be formed in the photoactive layer. Therefore, the pre-aggregation effect in solution is essential for realizing favorable morphology and thus high performance in NFA-OSCs. To investigate the aggregation behavior of PTVT-T in solution, the TD-Abs was measured by changing the temperature from 0°C to 120°C. As shown in Fig. 1c, with the temperature increasing, the whole absorption spectrum is gradually blue-shifted, and the absorption peak at long wavelength direction, at approximately 644 nm, decreases and finally disappears at 120°C. Such a strong aggregation effect is very similar to the other high-performance polymer donors [8,36] and has not been observed in most of the reported PTVs. It has been proven that the quantum efficiency of electroluminescence (EQE_EL_) plays a key role in determining the non-radiative energy loss and thus the open-circuit voltage (*V*_OC_) [37]. Herein, PTVT-T shows an EQE_EL_ value of 6 × 10^−4^ (Fig. S3b), which is comparable with the popularly used high performance polymers (in the range of 10^−5^–10^−3^) [38,39]. The cyclic voltammetry (CV) was then conducted to evaluate the energy levels of PTVT-T. As shown in Fig. S3c, the HOMO and LUMO levels are calculated to be −5.28 and −3.02 eV, respectively. The crystalline packing characteristics are investigated by grazing-incidence wide-angle X-ray scattering measurements (GIWAXS), and the 2D pattern, the corresponding in-plane (IP) and out-of-plane (OOP) profiles and parameters are shown in Fig. 1e and Table S8. As shown, a (010) reflection peak can be found at 1.75 Å^−1^ in OOP direction, indicating a face-on orientation with a π–π stacking distance of 3.59 Å. In addition, the (100) diffraction peak at 0.28 Å^−1^, which is mainly determined by the length of the alkyl chains of PTVT-T, can be observed in the neat film. We also investigated the surface morphology of the PTVT-T thin film by atomic force microscopy (AFM). As shown in Fig. 1f, PTVT-T film is quite smooth, i.e. with a mean square roughness (*R*_q_) of 0.77 nm, and fibrillar aggregations can be clearly observed, indicating that PTVT-T tends to form nanoscale aggregations from solution to solid state. From the collected information above, PTVT-T not only shows favorable opto-electrical properties that ensure light absorption capability and matched energy levels, but also presents a strong aggregation effect that will facilitate nano-scale phase separation in the PTVT-T:NFAs-blends.

**Figure 2.**
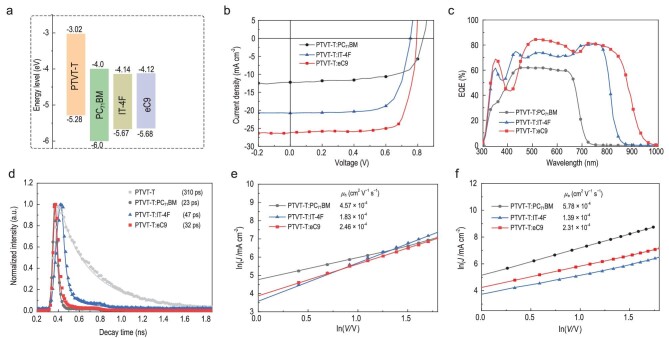
(a) Energy level diagram of PTVT-T, PC_71_BM, IT-4F and eC9. (b) *J-V* curves. (c) EQE spectra. (d) Normalized TRPL spectra. (e) The hole mobility and (f) electron mobility curves for PTVT-T:PC_71_BM-, PTVT-T:IT-4F- and PTVT-T:eC9-based devices.

In order to investigate the photovoltaic properties of PTVT-T, the OSCs with a structure of ITO/PEDOT:PSS/Active layer/PFN-Br/Al were fabricated and characterized by blending PTVT-T with PC_71_BM, IT-4F and eC9 (the structures are shown in Fig. S4), respectively, which are the most popular fullerene- and non-fullerene-based acceptors. The energy level diagrams of the materials used in the active layers are shown in Fig. 2a. The detailed device optimization process, including variation of host solvents, donor and acceptor (D/A) weight ratios, additives and thermal annealing methods, can be found in Figs S5–S9 and Tables S2–S6, and the current density voltage (*J-V*) curves as well as the corresponding photovoltaic parameters are provided in Fig. 2b and Table 2. As shown, a PCE of 7.25%, with a *V*_OC_ of 0.83 V, a short-circuit current density (*J*_SC_) of 12.78 mA cm^−2^ and a fill factor (FF) of 0.68, can be achieved in the PTVT-T:PC_71_BM-based OSC. While blending PTVT-T with the two representative NFAs, IT-4F and eC9, the *V*_OC_s of the corresponding devices are relatively lower than that of the PTVT-T:PC_71_BM-based device due to the lower-lying LUMO levels of the NFAs. However, the broader absorption, covering almost the whole visible region for the PTVT-T:IT-4F blend and even extending to the near-infrared region for the PTVT-T:eC9 blend, enable much higher *J*_SC_s in the PTVT-T:NFAs-based OSCs. Therefore, the PTVT-T:IT-4F-based OSC exhibits a PCE of 11.28%, with a *V*_OC_ of 0.75 V, a *J*_SC_ of 20.78 mA cm^−2^ and an FF of 0.72. A much higher PCE of 16.20% (*V*_OC_ = 0.79 V, *J*_SC_ = 26.22 mA cm^−2^, FF = 0.78) can be realized in the PTVT-T:eC9-based device, which is the highest value achieved in PTV derivatives-based OSCs.

**Table 2.** Photovoltaic parameters for the PTVT-T-based OSCs.

Active layer	*V*_OC_ (V)	*J*_SC_ (mA cm^−2^)	*J*_cal_^a^ (mA cm^−2^)	FF	PCE^b^ (%)
PTVT-T:PC_71_BM	0.83	12.78 (12.66 ± 0.01)	12.34	0.68	7.25 (6.09 ± 0.02)
PTVT-T:IT-4F	0.75	20.78 (20.75 ± 0.38)	20.66	0.72	11.28 (11.02 ± 0.06)
PTVT-T:eC9	0.79	26.22 (26.09 ± 0.01)	25.21	0.78	16.20 (16.01 ± 0.02)

^a^Integrated from EQE curves

^b^the average PCE values were obtained from over 20 devices.

The external quantum efficiencies (EQEs) and the absorption spectra of the three devices are demonstrated in Fig. 2c and Fig. S10. Comparing the EQE profiles for the three OSCs, the PTVT-T:PC_71_BM-based device shows a much narrower response range and relatively lower EQE peak value (∼60%) than the others. Benefitting from the red-shifted absorption of IT-4F and eC9, which possess complementary absorption spectra with PTVT-T in the visible-near infrared region, the corresponding devices show an obviously broadened response range of 300–850 nm and 300–950 nm, respectively. A much higher EQE with a peak value around 85% can be realized in the PTVT-T:eC9 device. The integrated current densities calculated from the EQE spectrum are 12.34, 20.66 and 25.21 mA cm^−2^ for PTVT-T:PC_71_BM-, PTVT-T:IT-4F- and PTVT-T:eC9-based OSCs, respectively, which are consistent with the *J*_SC_ values obtained from the *J*-*V* measurements. Additionally, time-resolved photoluminescence (TRPL) was measured to investigate the charge transfer between the donor and acceptors. The PL decay dynamics of the neat PTVT-T and three blend films are shown in Fig. 2d. The neat film of PTVT-T shows a relatively long fluorescence lifetime of 310 ps, which indicates a weak exciton recombination. The fluorescence lifetimes of the three blend films are 23 ps (PTVT-T:PC_71_BM), 47 ps (PTVT-T:IT-4F) and 32 ps (PTVT-T:eC9). The significantly quenched fluorescence suggests efficient charge transfer between PTVT-T and each of the acceptors.

Space-charge limited current (SCLC) measurement was employed to investigate the charge mobility (*μ*_h_ and *μ*_e_) of the PTVT-T neat film and the three blends. The *μ*_h_ of PTVT-T film is 5.78 × 10^−4^ cm^2^ V^−1^ s^−1^ (Fig. S11). As shown in Fig. 2e and f and Table S7, the carrier mobilities of the three blends are all at the same level, and the PTVT-T:eC9 film shows, relatively, well-balanced charge mobilities (*μ*_h_ of 2.46 × 10^−4^ cm^2^ V^−1^ s^−1^ and *μ*_e_ of 2.31 × 10^−4^ cm^2^ V^−1^ s^−1^) compared to those of the PTVT-T:PC_71_BM and PTVT-T:IT-4F blends. In addition, the relationship between photocurrent (*J*_ph_) and effective voltage (*V*_eff_) was measured and illustrated in Fig. S12a. The corresponding exciton dissociation probabilities

(*P*_diss_) were calculated to be 77%, 88% and 91% for PTVT-T:PC_71_BM-, PTVT-T:IT-4F- and PTVT-T:eC9-based devices, respectively, suggesting higher exciton dissociation and charge collection efficiencies in the PTVT-T:eC9-based device. We then measured the dependence of *J*_SC_ and *V*_OC_ on the light intensity so as to get more insight into the charge recombination kinetics of these three OSCs. The correlation of *J*_SC_ and the light intensity was described as *J*_ph_ ∝ P^s^, where the power-law exponent s represents the bimolecular recombination possibility. As displayed in Fig. S12b, the PTVT-T:eC9- and PTVT-T:IT-4F-based devices show the same s value of 0.98, which is slightly higher than that of the PTVT-T:PC_71_BM-based device (0.95), indicating that the bimolecular recombination in the three devices is quite low. Furthermore, the slope (s′) of Δ*V*_OC_ versus Δln(*P*_light_) (Fig. S12c), as the indication of the trap-state assisted charge recombination, is calculated to be 1.34, 1.36 and 1.22 kT q^−1^ for PTVT-T:PC_71_BM, PTVT-T:IT-4F and PTVT-T:eC9, respectively, showing that the PTVT-T:eC9-based device has the smallest trap-state assisted charge recombination, which partially contributes to its remarkable photovoltaic performance.

Photo-induced charger-carrier extraction in a linearly increasing voltage (photo-CELIV) measurement was conducted to further estimate the mobilities of faster carriers in the three OSCs. As depicted in Fig. S13, the photo-CELIV mobilities are calculated to be 1.35 × 10^–4^, 5.32 × 10^–5^ and 1.23 × 10^–4^ cm^2^ V^–1^ s^–1^ for PTVT-T:PC_71_BM-, PTVT-T:IT-4F- and PTVT-T:eC9-based devices, respectively.

**Figure 3.**
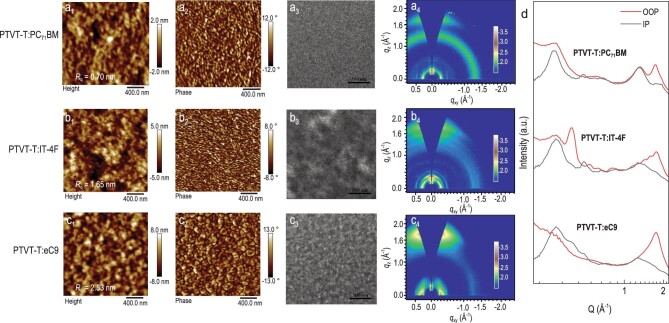
(a–c) AFM height (a_1_, b_1_ and c_1_) and phase (a_2_, b_2_ and c_2_) images, TEM images (a_3_, b_3_ and c_3_), 2D GIWAXS patterns (a_4_, b_4_ and c_4_) and (d) corresponding 1D cut-line profiles for PTVT-T:PC_71_BM, PTVT-T:IT-4F and PTVT-T:eC9 blends, respectively.

We investigated phase separation morphology of the three blends processed with the device fabrication conditions by AFM and transmission electron microscopy (TEM). As presented in Fig. 3a_1_, b_1_ and c_1_, the PTVT-T:eC9 shows slightly rougher surface morphology and higher *R*_q_ of 2.53 nm relative to PTVT-T:PC_71_BM (0.70 nm) and PTVT-T:IT-4F (1.65 nm) films. In the TEM images (Fig. 3a_3_, b_3_ and c_3_), fiber-like aggregations could be observed in PTVT-T:PC_71_BM and PTVT-T:eC9 blends, and the PTVT-T:IT-4F blend presents flake-like aggregates. Then, GIWAXS was employed to investigate the crystalline packing characteristics of the three blends. The 2D-GIWAXS patterns and the corresponding IP and OOP profiles are displayed in Fig. 3a_4_, b_4_, c_4_ and d, and the corresponding parameters and fitting curves are summarized in Table S8 and Fig. S14. As shown, the (100) diffraction peak at 0.28 Å^−1^, presented by the PTVT-T neat film, can be observed in all blends, and the neat film's face-on orientation is also retained in the three blends. Meanwhile, the (010) peaks were all measured at about 1.75 Å^−1^. Three separate diffraction (010) peaks can be observed in the PTVT-T:IT-4F film, which may be caused by the large differences in the domain size. Furthermore, the coherence lengths (CLs) of the PTVT-T in the three blend films were also estimated by fitting the (010) peaks. The CLs of the PTVT-T in PTVT-T:PC_71_BM, PTVT-T:IT-4F and PTVT-T:eC9 blends are 25.95, 39.24 and 26.95 Å, respectively. The excessive crystal size in PTVT-T:IT-4F may cause large phase separation, which is unfavorable for efficient exciton diffusion. The results are consistent with that of TRPL. Overall, these results imply that PTVT-T can form appropriate nano-scale networks with both fullerene- and non-fullerene-based acceptors, moreover, PTVT-T can guide in ‘face-on’ orientation and tight π-π stacking, which are favorable for charge transport.

**Figure 4.**
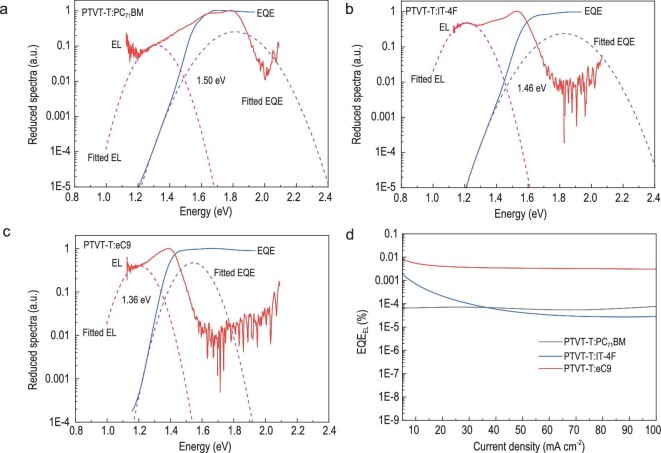
(a–c) The sEQE and EL curves; (d) EQE_EL_ of PTVT-T:PC_71_BM-, PTVT-T:IT-4F- and PTVT-T:eC9-based devices.

Highly sensitive EQE (sEQE) and EL spectra are collected to further investigate the energy loss of the three systems, as shown in Fig. 4. The corresponding three sources of *E*_loss_ parameters, which are defined in Equation 1, are summarized in Table S9.

(1) \begin{eqnarray*} E_{\textrm{loss}} &=& \Delta {E_1} + \Delta {E_2} + \Delta {E_3} \\ &=& ({E_g} - qV_{OC}^{SQ}) + (qV_{OC}^{SQ} - qV_{OC}^{\textrm{rad}}) \\ && +\,\, (qV_{OC}^{\textrm{rad}} - q{V_{OC}}). \end{eqnarray*}

Here, $\Delta {E_1}$ is unavoidable for any solar cells. $\Delta {E_2}$ is the radiative energy loss, which is closely related to the differences between the *E*_g_s and the energy of the charge-transfer state (*E*_CT_s) of the real devices [38]. $\Delta {E_3}$, which can be quantified as $- \frac{{kT}}{q}\ln {\rm{EQ}}{{\rm{E}}_{{\rm{EL}}}}$, plays a key role in determining *V*_OC_s of OSCs. The three systems show different $\Delta {E_1}$ values of 0.288 (PTVT-T:PC_71_BM), 0.270 (PTVT-T:IT-4F) and 0.263 eV (PTVT-T:eC9) due to the different optical band gaps. Through fitting the sEQE and the electroluminescence (EL) spectra, the *E*_CT_s of the devices based on PC_71_BM, IT-4F and eC9 are determined to 1.50, 1.46 and 1.36 eV, respectively. The relatively large energy offsets between *E*_g_s and *E*_CT_s lead to the radiative energy loss $\Delta {E_2}$ values of 0.328, 0.180 and 0.106 eV for PTVT-T:PC_71_BM-, PTVT-T:IT-4F- and PTVT-T:eC9-based devices, respectively. The EQE_EL_ of the three devices were measured to quantitatively calculate the non-radiative energy loss. The EQE_EL_ of the device based on PTVT-T:eC9 is 3.61 $\times $ 10^−5^, corresponding to a $\Delta {E_3}$ of 0.241 eV, which is lower than that of devices based on PTVT-T:PC_71_BM (0.334 eV) and PTVT-T:IT-4F (0.310 eV). The results also suggest that the device performance based on PTVT-T can be further improved by minimizing the energy loss, which is beyond the scope of this study. Herein, the stability of the encapsulated devices based on PTVT-T:NFAs were tested, and as shown in Fig. S15, the cells maintain over 80% of the initial PCE values after continuous illumination of AM 1.5G for ∼500 hours. The detailed experimental conditions and results are provided in the online Supplementary Data.

## CONCLUSION

In conclusion, we designed a PTV derivative polymer based on ester-substituted terthiophene and vinylene. The theoretical calculation shows that the ester group substituted TVT unit can overcome the intrinsic twisting effect by intramolecular O···H non-covalent interaction and help to obtain planar backbone structure, resulting in a strong aggregation behavior in solution state, which is one of the critical factors in achieving favorable phase separation morphology when blended with NFAs. In thin film, PTVT-T tends to form nano-scale aggregations and face-on orientation, contributing to effective intermolecular charge transportation. The devices based on PTVT-T and three representative fullerene and non-fullerene acceptors, including PC_71_BM, IT-4F and eC9, exhibit the PCEs of 7.25%, 11.28% and 16.20%, respectively, which are not only the best results for PTV-based polymers but also among the top values for OSCs based on other complex polymers. What is more, these encouraging results demonstrate that, with fine optimization of chemical structure, the polymer systems developed in the early stage of the OSC field may play a more important role in highly efficient OSCs due to their great advantage in low-cost, which is a critical issue for future OSC industrialization.

## Supplementary Material

nwab031_Supplemental_File
