# Supplementary material for: Molecular design revitalizes the low-cost PTV-polymer for highly efficient organic solar cells
Source: Natl Sci Rev. 2021 Feb 12;8(8):nwab031. doi: 10.1093/nsr/nwab031 (PMC8966978; doi:10.1093/nsr/nwab031)
Supplement: nwab031_Supplemental_File [file nwab031_supplemental_file.docx]

**Supporting Information**

Molecular Design Revitalizes the Low-cost PTV-Polymer for Highly Efficient Organic Solar Cells

Junzhen Ren,^1,2^ Pengqing Bi,^2^ Jianqi Zhang,^3^ Jiao Liu,^1,2^ Jingwen Wang,^2^ Ye Xu,^2^ Zhixiang Wei,^3^ Shaoqing Zhang^1,2^* and Jianhui Hou^1,2*^

**Materials**

Compound 1, 2, 3, T-Sn, PC_71_BM, IT-4F and eC9, were purchased from Solarmer Materials Inc. Other reagents and solvents were purchased from Innochem, J&K Scientific and other commercial sources. All chemicals were used as received without further purification. V-Sn, compound 4, 5 and M1 were synthesized as the following procedure:

***(E)-1,2-bis(tributylstannyl)ethene (V-Sn)***

Tributylethynylstannane (1) (60 g, 190 mmol) and tributyltin hydride (2) (57.9 g, 199 mmol) were put into a 500mL flask, and 450 mg of 2,2’-azobis(2-methylpropionitrile) (AIBN) was added. Under the protection of nitrogen, the mixture was heated to 100 °C for 6 h. Distillation (170 °C, 40 Pa) yielded 104.8 g (91%) of compound V-Sn ethylene as a clear, colorless oil.

^1^H NMR (400 MHz, CDCl_3_) δ(ppm): 6.88 (s, 2H), 1.59-1.42 (m, 12 H), 1.39-1.25 (m,12 H), 0.96-0.85 (m, 30 H).

***2-butyloctyl 2-bromothiophene-3-carboxylate (4)***

2-bromothiophene-3-carboxylic acid (3) (3.00 g, 14.49 mmol), DCC (4.48 g, 21.74 mmol) and DMAP (1.06 g, 8.69 mmol) was dissolved in 20 mL of dry dichloromethane under inert atmosphere protection, and 2-butyloctanol（4.05 g, 21.74 mmol) was added into the mixture slowly. After stirred at room temperature for 24 h, the organic layer was washed with water, and then dried over anhydrous MgSO_4_. The organic layer was concentrated by vacuum evaporation, and the residue was purified by column chromatography on silica gel using petroleum ether: dichloromethane (2:1) as eluent to give compound 4 (4.84 g, yield = 89%) as a colorless liquid.

^1^H NMR (400 MHz, CDCl_3_) δ(ppm): 7.36 (d, 1H), 7.21 (d, 1H), 4.20 (d, 2H), 1.74 (m, 1H), 1.40-1.28 (m, 16H), 0.90 (m, 6H).

***Bis(2-butyloctyl) 2,2'-(ethene-1,2-diyl)(E)-bis(thiophene-3-carboxylate) (5)***

Compound 4 (3.97 g, 10.58 mmol), V-Sn (2.79 g, 4.60 mmol) and Pd(PPh_3_)_4_ (150 mg, 0.13 mmol) was mixed in 20 mL of anhydrous toluene and 2 ml of anhydrous DMF, and the solution was purged by argon for 15 minutes. Then the mixture was stirred at 110 °C overnight. After cooled to room temperature, the crude product was concentrated under reduced pressure and further purified by column chromatography on silica gel using petroleum ether: dichloromethane (1:1) as eluent. Finally, compound 5 was obtained as light yellow solid (2.33 g, yield = 82%) by recrystallization from ethanol. MS (MALDI-TOF): m/z 639.2 (M+Na).

^1^H NMR (400 MHz, CDCl_3_) δ(ppm): 8.19 (s, 2H), 7.44 (d, 2H), 7.13 (d, 2H), 4.22 (d, 4H), 1.78 (m, 2H), 1.32-1.27 (m, 32H), 0.89 (m, 12H).

^13^C NMR (400 MHz, CDCl_3_) δ(ppm): 163.54, 148.58, 130.18, 129.32, 124.43, 123.32, 67.63, 37.51, 31.83, 31.53, 31.19, 29.64, 29.03, 26.79, 23.01, 22.66, 14.08, 14.05.

***Bis(2-butyloctyl) 2,2'-(ethene-1,2-diyl)(E)-bis(5-bromothiophene-3-carboxylate) (M1)***

Compound 5 (1.05 g, 1.70 mmol) was dissolved in a mixed solvent of CHCl_3_ (50 mL) and trifluoroacetic acid (10 mL) at 0 °C in dark, and then N-Bromosuccinimide (NBS) (665 mg, 3.74 mmol) was added in the solution in several portions. The reaction mixture was stirred overnight and poured into water. The organic layer was then concentrated by rotary evaporation. The residue was preliminarily purified by column chromatography on silica gel using petroleum ether:dichloromethane (1:1), then followed by the recrystallization from ethanol. The compound M1 was obtained as light yellow solid (1.22 g, yield = 93%). MS (MALDI-TOF): m/z 797.1 (M+Na).

^1^H NMR (400 MHz, CDCl_3_) δ(ppm): 8.01 (s, 2H), 7.37 (s, 2H), 4.20 (d, 4H), 1.75 (t, 2H), 1.38-128 (m, 32H), 0.89 (m, 12H).

^13^C NMR (400 MHz, CDCl_3_) δ(ppm): 162.29, 149.43, 132.58, 129.79, 123.92, 111.25, 68.01, 37.48, 31.85, 31.47, 31.15, 29.63, 29.04, 26.78, 23.01, 22.66, 14.10, 14.07.

***Polymerization for PTVT-T***

2,5-bis(trimethylstannyl)thiophene (T-Sn) (122.9 mg, 0.30 mmol) and M1 (231.6 mg, 0.30 mmol) were dissolved into a mixed solvent of anhydrous chlorobenzene (CB) (10 mL) and DMF (1 mL). The reaction mixture was flushed with argon for 5 min, and 10 mg of Pd(PPh_3_)_4_ was added into the solution subsequently. The mixture was purged with argon for another 15 min and allowed to stir at 115 °C for 12 h under argon atmosphere. After that, the reactant was cooled down to room temperature, and the polymer was precipitated into 100 mL of methanol, filtrated and further purified by silica gel column chromatography by using chlorobenzene as eluent. The polymer was precipitated again in 100 mL of methanol and dried by vacuum, with a yield of 85%.

C_40_H_58_O_4_S_3_ (PTVT-T): Calculated C: 68.72 H: 8.36. Found C: 68.92 H: 8.09.

**DFT calculation**

The molecular geometries were optimized by Gaussian 09 with a functional of B3LYP and a basis set of 6- 31G(d,p).[1] To save time, the alkyls in the ester groups were replaced with methyl groups.

**Device Fabrication**

The devices were fabricated with a conventional architecture of ITO/PEDOT:PSS/Active layer/PFN-Br/Al. The ITO-coated glass substrates were washed ultrasonically in water/detergent, water, acetone, and isopropanol in sequence for 15 min. After oxygen plasma treatment for 20 min, the substrate was spin-coated with a thin layer of PEDOT:PSS (30 nm) and then dried under argon at 120 °C for 20 min. The PTVT-T:eC9 (1:1.2, w/w) blends was dissolved in chloroform with a concentration of 6 mg m^-l^ (for polymer), with DPE (0.5% by volume) as additive. The solution was stirred for 5 hours at 40 °C and then spin-coated on the surface of PEDOT:PSS layer in nitrogen-based atmosphere glove box, and the optimal film of the active layer is ca. 100 nm. The active layer was then thermal annealed for 10 min at 80 °C. 10 nm thick of PFN-Br was spin-coated on the top of the active layers at 3000 rpm for 30 s. Finally, 100 nm thick of Al layer were deposited onto the active layer under high vacuum of ~3×10^-4^ Pa. The overlapping area between the cathode and anode was 4 mm^2^. The *J-V* curves were measured under 100 mW cm^-2^ of the standard AM 1.5G spectrum.

Optimal fabricating condition was acquired based on PTVT-T:PC_71_BM, and then

PTVT-T:IT-4F based devices adopted the same condition directly without special optimization.

**Instruments and Measurements**

Gel permeation chromatography (GPC) was performed to provide the molecular weight and the polydispersity (PDI) by using trichlorobenzene as eluent at 140 °C on a PL GPC-220 instrument. The UV-visible absorption spectroscopy measurements were conducted on a Hitachi UH4150 spectrophotometer. Thermo gravimetric analysis (TGA) measurements were carried out on TGA-2050 from TA Instruments, Inc. The cyclic voltammograms (CV) measurements were recorded on a Zahner IM6e electrochemical workstation by using glassy carbon discs as the working electrode, Pt wire as the counter electrode, Ag/AgCl electrode as the reference electrode with a scanning rate of 20 mV s^-1^ in a 0.1 M tetrabutylammonium hexafluorophosphate (Bu_4_NPF_6_) solution, and the potential of Ag/Ag+ reference electrode was internally calibrated by using ferrocene/ferroncenium (Fc/Fc+) as the redox couple. The *J−V* measurement was performed via the solar simulator (SS-F5-3A, Enlitech) along with AM 1.5G spectra whose intensity was calibrated by the certified standard silicon solar cell (SRC-2020, Enlitech) at 100 mw cm^-2^. The external quantum efficiency (EQE) was measured by Solar Cell Spectral Response Measurement System QE-R3011 (Enli Technology Co., Ltd.). Fluorescence decay dynamics were recorded by TCSPC (Becker&Hickl, SPC-150). The excitation wavelength is 500 nm with a laser power of *ca.* 20 μJ cm^‑2^. The laser spot diameter is 2 μm. The photo-CELIV measurements reported were performed by the all-in-one characterization platform Paios developed and commercialized by Fluxim AG, Switzerland. The tapping mode Atom Force Microscopy (AFM) measurements were performed on a Bruker Nanoscope V AF microscope. Transmission electron microscopy (TEM) images were obtained on a Tecnai G2 F20 U-TWIN TEM instrument. GIWAXS measurements were performed on a XEUSS SAXS/WAXS system (XENOCS, France) at the National Center for Nanoscience and Technology (NCNST, Beijing).  Highly sensitive EQE (s-EQE) was measured using an integrated system (PECT-600, Enlitech). External quantum efficiency of electroluminescence (EQE_EL_) and electroluminescence (EL) spectra were collected by applying external voltage (0-5 V) through the devices (ELCT-3010, Enlitech). The devices used for EQE_EL_ and EL spectra measurements were fabricated according to the optimized conditions. The detailed calculations of energy loss follow the reported work[2] and described as follows:

$V_{OC}^{SQ}=\frac{kT}{q}\ln(\frac{J_{SC}}{J_{0}^{SQ}}+1)=\frac{kT}{q}\ln(\frac{q\cdot\int_{0}^{\infty} {EQE}_{PV}\left( E \right)\cdot\emptyset_{AM1.5}\left( E \right)dE}{q\cdot\int_{E_{gap}}^{\infty} \emptyset_{BB}\left( E \right)dE}+1)$ (1)

$V_{OC}^{rad}=\frac{kT}{q}\ln(\frac{J_{SC}}{J_{0}^{rad}}+1)=\frac{kT}{q}\ln(\frac{q\cdot\int_{0}^{\infty} {EQE}_{PV}\left( E \right)\cdot\emptyset_{AM1.5}\left( E \right)dE}{q\cdot\int_{0}^{\infty} {EQE}_{PV}\left( E \right)\cdot\emptyset_{BB}\left( E \right)dE}+1)$ (2)

$\emptyset_{BB}\left( E \right)=\frac{2\pi}{h^{3}c^{2}}E^{2}exp\left( -\frac{E}{KT} \right)$ (3)

${\Delta E}_{1}=E_{g}-V_{oc}^{SQ}$ (4)

${\Delta E}_{2}=V_{OC}^{SQ}-V_{oc}^{rad}$ (5)

$\Delta E_{3}=-\frac{kT}{q}\ln{EQE}_{EL}$ (6)

The stability of the PTVT-T:NFAs-based OSCs were measured by a Muti-channel Thin Film Photovoltaic Performance Decay Testing System (Model: PVLT-6001M-16A) with tracing the maximum output point. The temperature of the cell surface during the test is 50±5 oC due to the heating effect of the light.


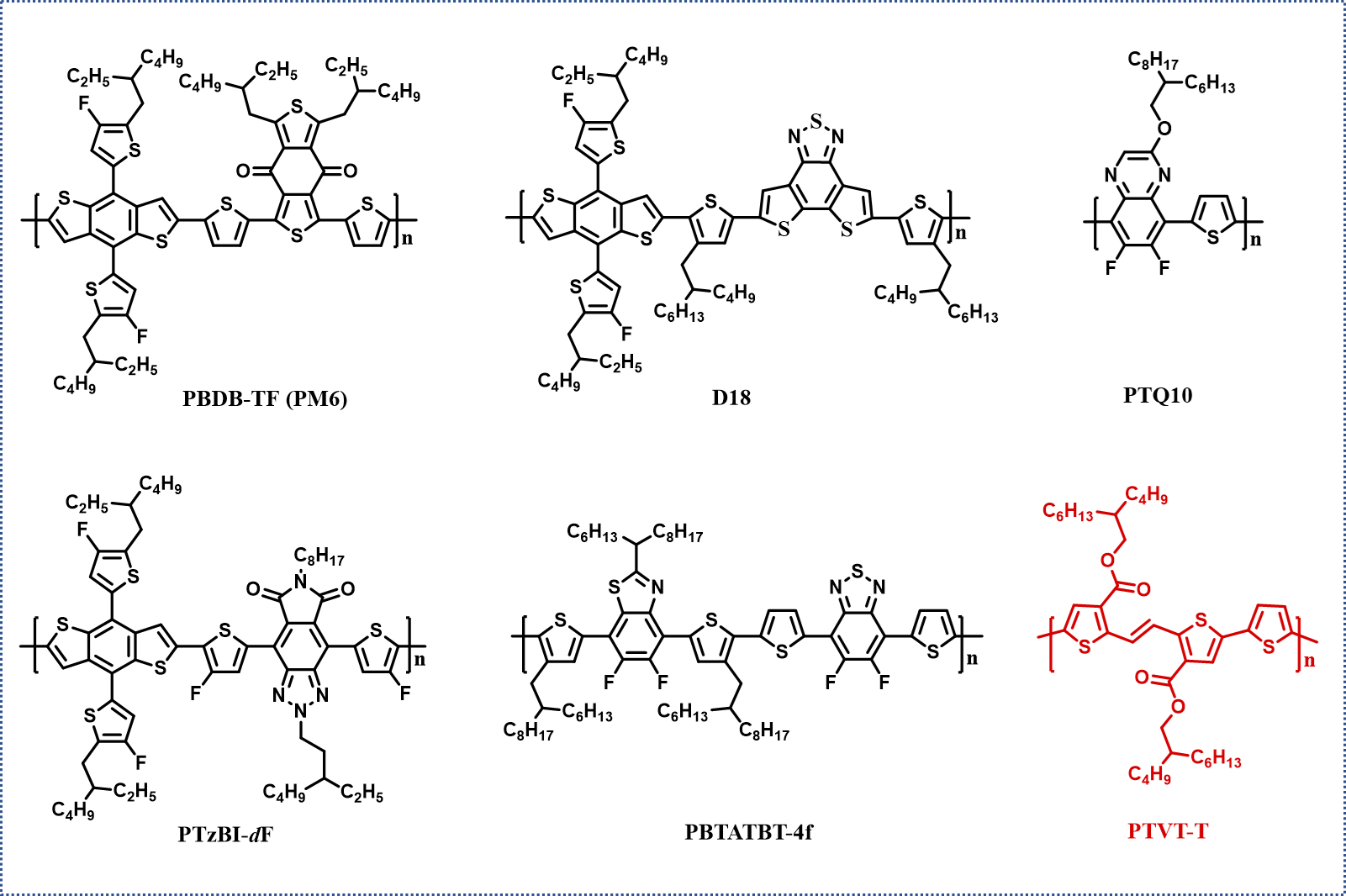


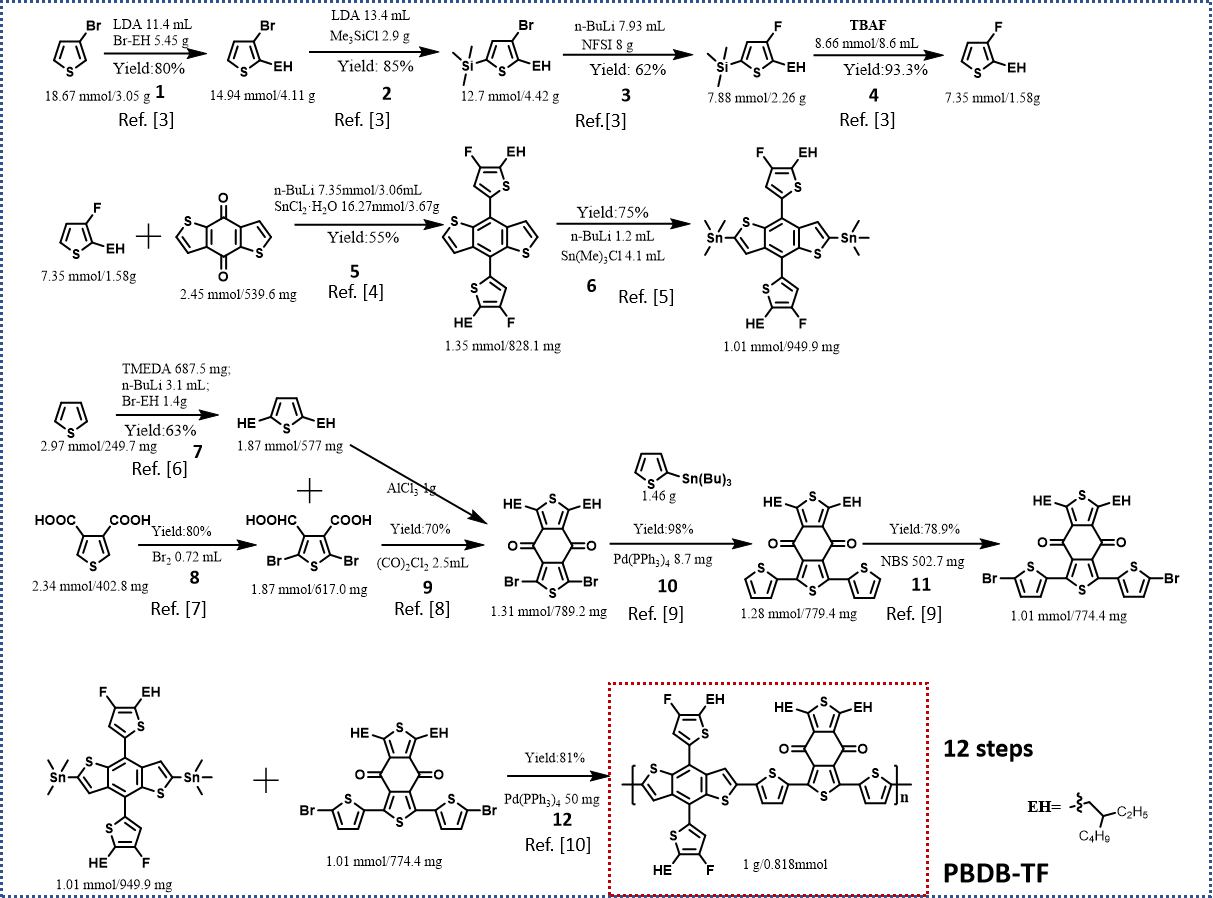


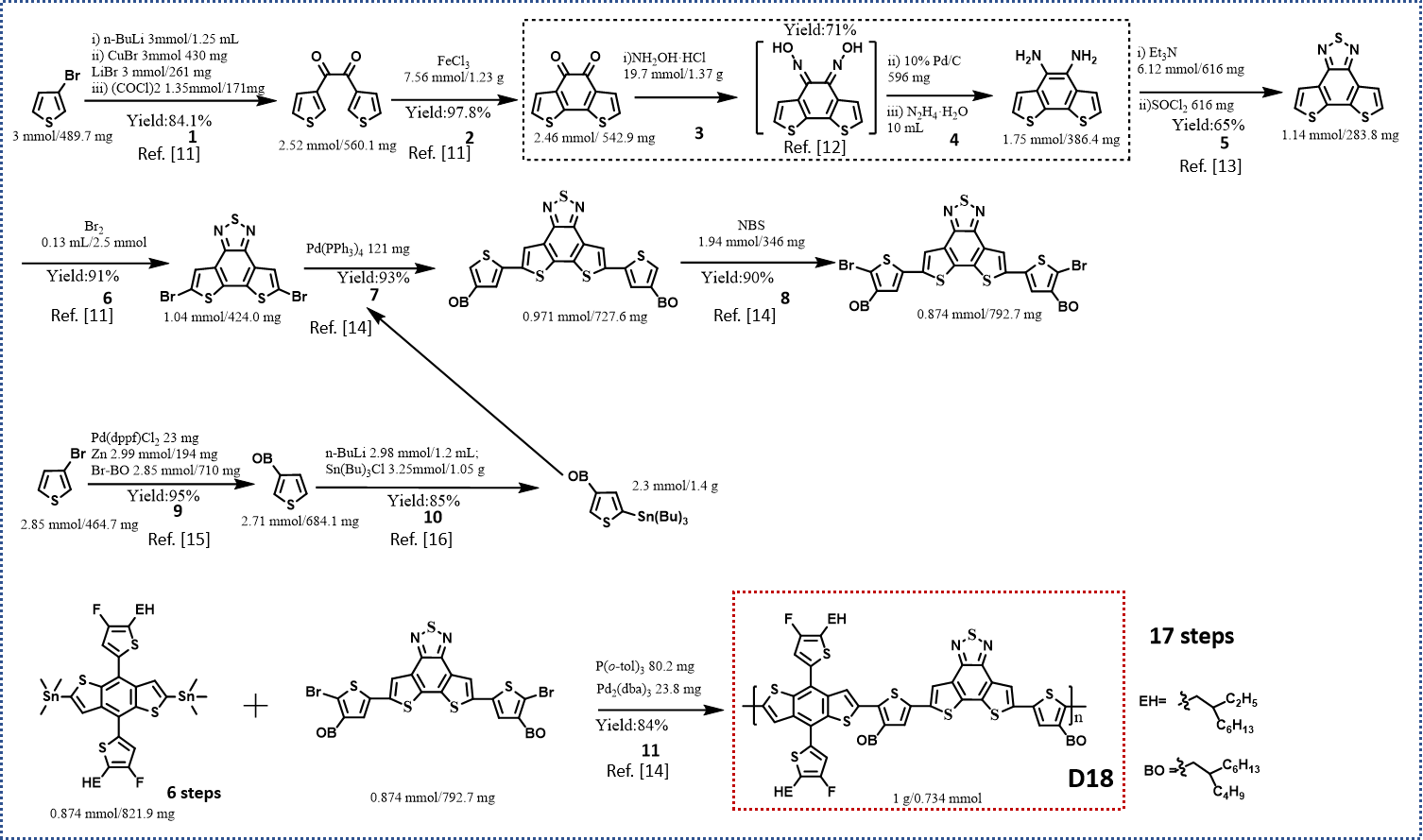


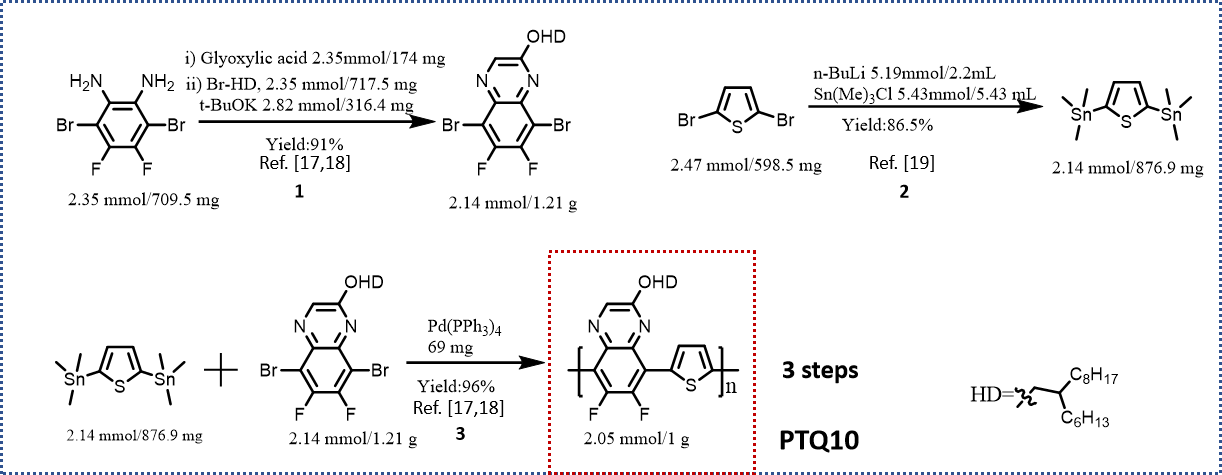


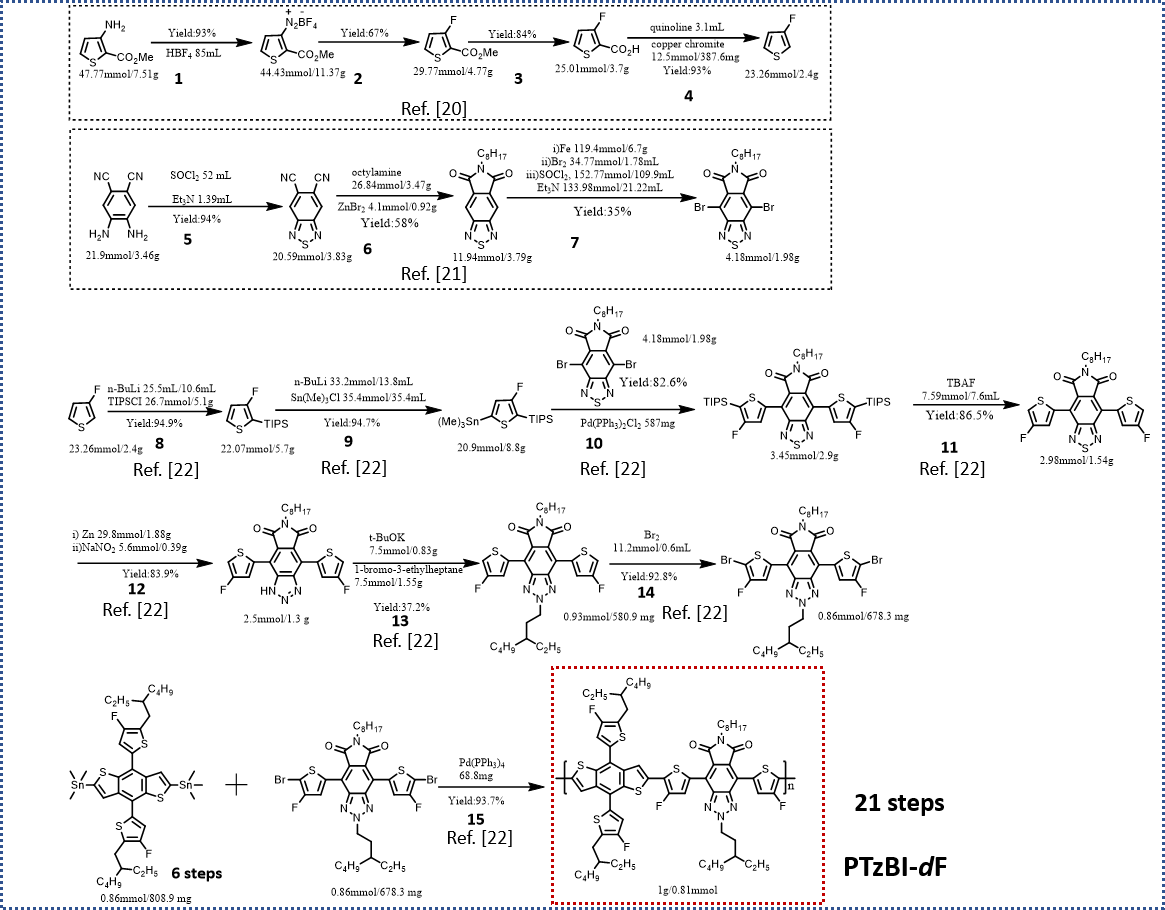


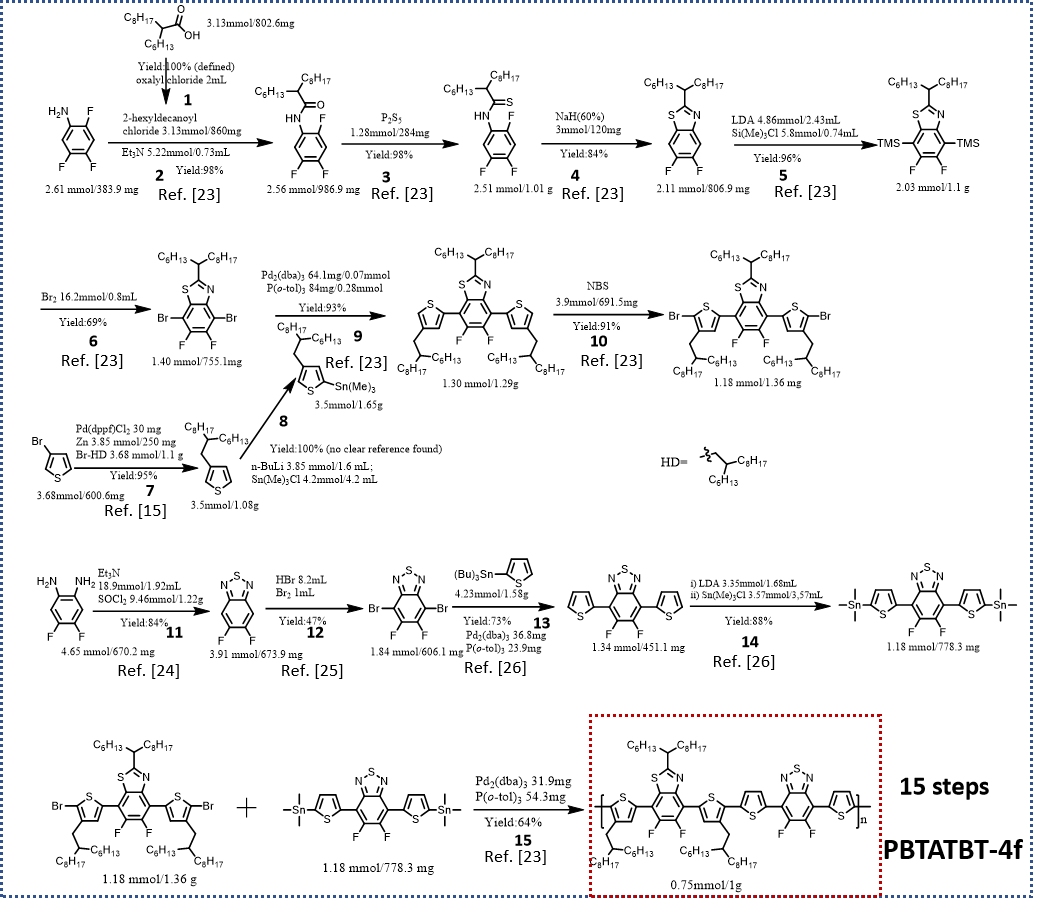


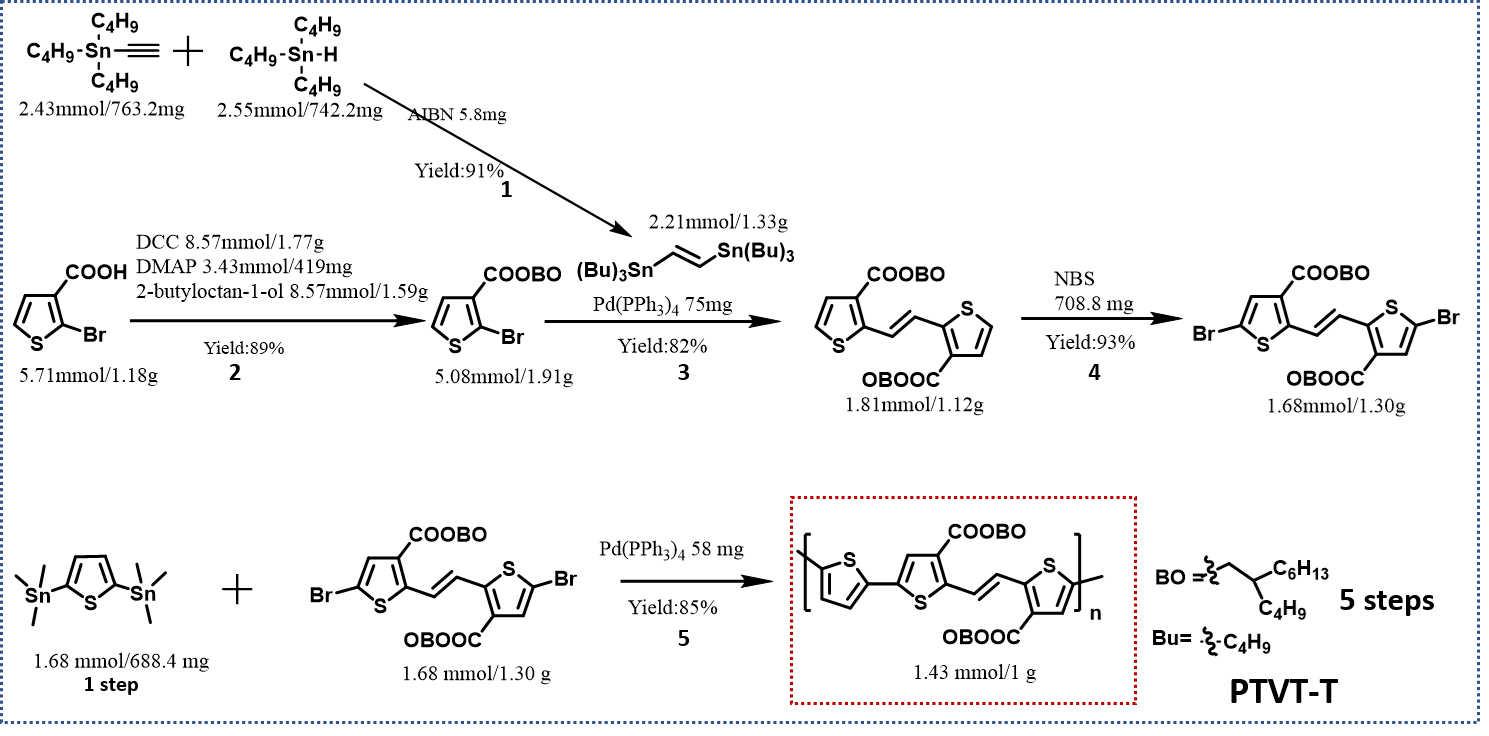


**Figure S1.** The detailed synthetic route and the yield of each step of typical high-performance polymer donors and PTVT-T. Note that the initial raw material here are defined as one can be bulkily purchased in hectograms or kilograms (excluding the catalysts). In order to ensure the accuracy of statistical data, each step of synthesis is referred to designated references, and the yield of each step is one of the highest yields available on Reaxys or Scifinder. In addition, the references are preferred to the work that firstly reported the corresponding polymer.

**Table S1.** The MOC evaluation of typical high-performance polymer donors (PCE over 16%) and PTVT-T.^a^

| **PBDB-TF (1g)** [3-10] | | | |
| --- | --- | --- | --- |
| Donor monomer | | | |
| Reagent | Quantity | Unit (source) ^b,c^ (By November 2020) | Cost ($) |
| 3-bromothiophene | 3.05 g | 93.9$/500 g (J&K) | 0.57 |
| LDA | 11.4+13.4 mL | 76.2$/500 mL, 2M (J&k) | 3.78 |
| Br-EH | 5.45 g | 89.8$/500 g (J&k) | 0.98 |
| Si(Me)_3_Cl (ρ=0.86g/mL) | 2.9 g | 46.7$/500 mL (J&K) | 0.31 |
| n-BuLi | 7.93+3.06+1.4mL | 43.6$/800 mL, 2.4M (J&K) | 0.68 |
| NFSI | 8 g | 92.9$/500 g (J&K) | 1.49 |
| TBAF | 8.6 mL | 239.7$/500 mL, 1M (J&K) | 4.12 |
| benzo[1,2-b:4,5-b']dithiophene-4,8-dione | 539.6 mg | 149.2$/10 g (J&K) | 8.04 |
| SnCl_2_·H_2_O | 3.67 g | 32.6$/500 g (J&K) | 0.24 |
| Sn(Me)_3_Cl | 4.9 mL | 220.3$/100 mL,1M (J&K) | 10.79 |
| Total Cost | | | 31 |
|  | | | |
| Acceptor monomer | | | |
| Reagent | Quantity | Unit(source) (By November 2020) | Cost ($) |
| Br-EH | 1.4 g | 89.8$/500 g (J&K) | 0.25 |
| n-BuLi | 3.1 mL | 43.6$/800 mL,2.4M (J&K) | 0.17 |
| thiophene | 249.7 mg | 39.2$/500 g (J&k) | 0.02 |
| TMEDA (ρ=0.77g/mL) | 690.3 mg | 33.3$/500 mL (J&K) | 0.06 |
| thiophene-3,4-dicarboxylic acid | 402.8 mg | 1357$/100 g (J&K) | 5.47 |
| Br_2_ (ρ=3.119g/mL) | 0.72 mL | 28.5$/250 g (Alfa Aesar) | 0.26 |
| AlCl_3_ | 1 g | 37.8$/100 g (TCI) | 0.38 |
| Oxalyl chloride (ρ=1.5g/mL) | 2.5 mL | 64.8$/100 g (J&K) | 2.43 |
| tributyl(thiophen-2-yl)stannane (ρ=1.18g/mL) | 1.46 g | 954.5$/250 mL (Sigma-Aldrich) | 4.72 |
| Pd(PPh_3_)_4_ | 8.7 mg | 36.4$/1 g (J&K) | 0.32 |
| NBS | 478.5 mg | 95.3$/1kg (J&K) | 0.05 |
| Total Cost |  |  | 14.13 |
|  | | | |
| Polymerization (1g) | | | |
| Reagent | Quantity | Unit(source) (By November 2020) | Cost ($) |
| Pd(PPh_3_)_4_ | 50 mg | 36.4$/1 g (J&K) | 1.82 |
| **Overall cost for 1g PBDB-TF** | | | **46.9** |
|  | | | |
| **D18 (1g)**[11-16] | | | |
| Donor monomer | | | |
| Reagent | Quantity | Unit(source) (By November 2020) | Cost ($) |
|  | 821.9 mg | 32.64$/1g (calculated from donor monomer cost of PBDB-TF) | 26.83 |
|  | | | |
| Acceptor Monomer | | | |
| Reagent | Quantity | Unit(source) (By November 2020) | Cost ($) |
| 3-bromothiophene | 489.7 mg | 93.9$/500g (J&K) | 0.09 |
| n-BuLi | 1.25 mL | 43.6$/800mL,2.4M (J&K) | 0.07 |
| CuBr | 430 mg | 189.5$/1kg (J&K) | 0.08 |
| LiBr | 261mg | 155.1$/2.5kg (J&K) | 0.02 |
| Oxalyl chloride | 171 mg | 64.8$/100g (J&K) | 0.11 |
| FeCl_3_ | 1.23 g | 24.5$/1kg (J&K) | 0.03 |
| NH_2_OH·HCl | 1.37 g | 19.1$/1kg (J&K) | 0.03 |
| 10% Pd/C | 596 mg | 117$/10g (J&K) | 6.97 |
| N_2_H_4_·H_2_O (ρ=1.03g/mL) | 10 mL | 279.4$/500g (Sigma-Aldrich) | 5.5 |
| Et_3_N (ρ=0.73g/mL) | 616 mg | 67.8$/1L (J&K) | 0.07 |
| thionyl chloride (SOCl_2_) | 616 mg | 109.5$/500g (Alfa Aesar) | 0.13 |
| Br_2_ (ρ=3.119g/mL) | 0.13 mL | 28.5$/250 g (Alfa Aesar) | 0.05 |
| Pd(PPh_3_)_4_ | 121 mg | 36.4$/1g (J&K) | 4.4 |
| NBS | 346 mg | 95.3$/1kg (J&K) | 0.03 |
| Pd(dppf)Cl_2_ | 23 mg | 55$/1g (J&K) | 1.27 |
| Zn | 194 mg | 96.6$/1kg (Sigma-Aldrich) | 0.02 |
| Br-BO | 710 mg | 453$/25g (TCI) | 12.9 |
| n-BuLi | 1.2 mL | 43.6$/800 mL,2.4M (J&K) | 0.07 |
| Sn(Bu)_3_Cl | 1.05 g | 213$/500g (J&K) | 0.45 |
| Total cost | | | 32.29 |
|  | | | |
| Polymerization (1g) | | | |
| Reagent | Quantity | Unit(source) (By November 2020) | Cost ($) |
| P(*o*-tol)_3_ | 80.2 mg | 8.2$/1g (J&K) | 0.66 |
| Pd_2_(dba)_3_ | 23.8 mg | 150$/1g (J&K) | 3.57 |
| **Overall cost for 1g D18** |  |  | **63.4** |
|  | | | |
| **PTQ10 (1g)**[17-19] | | | |
| Donor Monomer | | | |
| Reagent | Quantity | Unit(source) (By November 2020) | Cost ($) |
| 2,5-dibromothiophene | 598.5mg | 86.7$/500g (J&K) | 0.1 |
| n-BuLi | 2.2 mL | 43.6$/800 mL,2.4M (J&K) | 0.12 |
| Me_3_SnCl | 5.43 mL | 220.3$/100 mL,1M (J&K) | 11.96 |
| Total cost |  |  | 12.18 |
|  | | | |
| Acceptor Monomer | | | |
| Reagent | Quantity | Unit(source) (By November 2020) | Cost ($) |
| 3,6-dibromo-4,5-difluorobenzene-1,2-diamine | 709.5 mg | 946.9$/5g (J&K) | 134.4 |
| Glyoxylic acid | 174 mg | 52.9$/500g (J&K) | 0.02 |
| 7-(bromomethyl)pentadecane | 17.5 mg | 113.6$/100g (J&K) | 0.02 |
| t-BuOK | 316.4mg | 47.2$/500g (J&K) | 0.03 |
| Total cost | | | 134.47 |
|  | | | |
| Polymerization (1g) | | | |
| Reagent | Quantity | Unit(source) (By November 2020) | Cost ($) |
| Pd(PPh3)4 | 69mg | 36.4$/ 1 g (J&K) | 2.51 |
| **Overall cost for 1g PTQ10** | | | **149.2** |
|  | | | |
| **PTzBI-*d*F**[20-22] | | | |
| Donor monomer | | | |
| Reagent | Quantity | Unit(source) (By November 2020) | Cost ($) |
|  | 808.9mg | 32.64$/1g (calculated from donor monomer cost of PBDB-TF) | 26.4 |
|  | | | |
| Acceptor monomer | | | |
| Reagent | Quantity | Unit(source) (By November 2020) | Cost ($) |
| methyl 3-aminothiophene-2-carboxylate | 7.51g | 64.39$/500g (J&K) | 0.97 |
| HBF_4_ (ρ=1.84g/mL) | 85mL | 11.36$/500g (J&K) | 3.55 |
| quinoline (ρ=1.09g/mL) | 3.1mL | 53.33$/500g (J&K) | 0.36 |
| copper chromite | 387.6mg | 104.5$/100g (J&K) | 0.41 |
| 4,5-diaminophthalonitrile | 3.46g | 472.7$/5g (J&K) | 327.1 |
| SOCl_2_ | 52mL+109.9mL | 109.5$/500 g (Alfa Aesar) | 58.15 |
| Et_3_N | 1.39mL+21.22mL | 67.8$/1L (J&K) | 1.53 |
| octylamine | 3.47g | 131.4$/500g (J&K) | 0.91 |
| ZnBr_2_ | 0.92g | 17.4$/500g (J&K) | 0.03 |
| Fe | 6.7g | 90.7$/1kg (J&K) | 0.61 |
| Br_2_ (ρ=3.119g/mL) | 1.78mL+0.6mL | 28.5$/250 g (Alfa Aesar) | 0.85 |
| n-BuLi | 10.6mL+13.8mL | 43.6$/800 mL,2.4M (J&K) | 1.33 |
| TIPSCl | 5.1g | 94.69$/500g (J&K) | 0.97 |
| Sn(Me)_3_Cl | 35.4mL | 220.3$/100 mL,1M (J&K) | 77.98 |
| Pd(PPh_3_)_2_Cl_2_ | 587mg | 55$/1g (J&K) | 32.29 |
| TBAF | 7.6mL | 239.7$/ 500 mL,1M (J&K) | 3.64 |
| Zn | 1.88g | 96.6$/1kg (Sigma-Aldrich) | 0.18 |
| t-BuOK | 0.83g | 47.2$/500g (J&K) | 0.08 |
| Total cost |  |  | 510.94 |
|  | | | |
| Polymerization (1g) | | | |
| Reagent | Quantity | Unit(source) (By November 2020) | Cost ($) |
| Pd(PPh_3_)_4_ | 68.8mg | 36.4$/ 1 g (J&K) | 2.5 |
| **Overall cost for 1g PTzBI-*d*F** |  |  | **539.8** |
|  | | | |
| **PBTATBT-4f**[15, 23-26] | | | |
| Donor Monomer | | | |
| Reagent | Quantity | Unit(source) (By November 2020) | Cost ($) |
| 2,4,5-trifluoroaniline | 383.9 mg | 98.5$/100g (J&K) | 0.38 |
| 2-hexyldecanoic acid | 802.6 mg | 81.8$/100g (J&K) | 0.66 |
| Oxalyl chloride (ρ=1.5g/mL) | 2mL | 64.8$/100 g (J&K) | 1.94 |
| Et_3_N | 0.73mL | 67.8$/1L (J&K) | 0.05 |
| P_2_S_5_ | 284 mg | 110.2$/500g (Acros) | 0.06 |
| NaH(60%) | 120mg | 100.8$/500g (TCI) | 0.02 |
| LDA | 2.43mL | 76.2$/500 mL,2M (J&k) | 0.37 |
| Si(Me)_3_Cl | 0.74mL | 46.7$/500 mL (J&K) | 0.07 |
| Br_2_ (ρ=3.119g/mL) | 0.8mL | 28.5$/250 g (Alfa Aesar) | 0.28 |
| P(*o*-tol)_3_ | 84mg | 8.2$/1g (J&K) | 0.69 |
| Pd_2_(dba)_3_ | 64mg | 150$/1g (J&K) | 9.6 |
| NBS | 691.5mg | 95.3$/ 1kg (J&K) | 0.07 |
| Pd(dppf)Cl_2_ | 30mg | 55$/1g (J&K) | 1.65 |
| Zn | 250mg | 96.6$/1kg (Sigma-Aldrich) | 0.02 |
| 7-(bromomethyl)pentadecane | 1.1g | 113.6$/100 g (J&K) | 1.25 |
| 3-bromothiophene | 600.6mg | 161.7$/500 g (J&K) | 0.19 |
| n-BuLi | 1.6mL | 43.6$/800 mL,2.4M (J&K) | 0.09 |
| Sn(Me)_3_Cl | 4.2mL | 220.3$/100 mL,1M (J&K) | 9.25 |
| Total Cost |  |  | 26.64 |
|  | | | |
| Acceptor Monomer | | | |
| Reagent | Quantity | Unit(source) By November 2020 | Cost ($) |
| 4,5-difluorobenzene-1,2-diamine | 670.2 mg | 406.4$/100g (J&K) | 2.73 |
| Et_3_N | 1.92mL | 67.8$/1L (J&K) | 0.13 |
| SOCl_2_ | 1.22g | 109.5$/500 g (Alfa Aesar) | 0.27 |
| HBr | 8.2mL | 58.6$/500 mL (J&K) | 0.96 |
| Br_2_ (ρ=3.119g/mL) | 1mL | 28.5$/250 g (Alfa Aesar) | 0.36 |
| tributyl(thiophen-2-yl)stannane (ρ=1.18g/mL) | 1.58g | 954.5$/250 mL (Sigma-Aldrich) | 5.11 |
| P(*o*-tol)_3_ | 23.9 mg | 8.2$/1g (J&K) | 0.20 |
| Pd_2_(dba)_3_ | 36.8 mg | 150$/1g (J&K) | 5.52 |
| LDA | 1.68 mL | 76.2$/500 mL,2M (J&k) | 0.26 |
| Sn(Me)_3_Cl | 3.57 mL | 220.3$/100 mL,1M (J&K) | 7.86 |
| Total Cost |  |  | 23.4 |
|  |  |  |  |
| Polymerization (1g) | | | |
| Reagent | Quantity | Unit(source) (By November 2020) | Cost ($) |
| P(*o*-tol)_3_ | 54.3 mg | 8.2$/1g (J&K) | 0.45 |
| Pd_2_(dba)_3_ | 31.9 mg | 150$/1g (J&K) | 4.79 |
| **Overall cost for 1g PBTATBT-4f** |  |  | **55.3** |
|  | | | |
| **PTVT-T** | | | |
| Donor Monomer | | | |
| Reagent | Quantity | Unit(source) (By November 2020) | Cost ($) |
|  | 688.4mg | 13.89$/1g (calculated from donor monomer cost of PTQ10) | 9.56 |
|  | | | |
| Acceptor Monomer | | | |
| Reagent | Quantity | Unit(source) (By November 2020) | Cost ($) |
| tributyl(ethynyl)stannane | 763.2mg | 115.1$/25g (J&K) | 3.51 |
| AIBN | 5.8mg | 23.48$/500g (J&K) | 0.0003 |
| tributylstannane | 742.7mg | 200.2$/500g (J&K) | 0.29 |
| 2-bromothiophene-3-carboxylic acid | 1.18g | 318.2$/25g (J&K) | 15.02 |
| DCC | 1.77g | 25.8$/500g (J&K) | 0.09 |
| DMAP | 419mg | 43.2$/500g (J&K) | 0.04 |
| 2-butyloctan-1-ol (ρ=0.833g/mL) | 1.59g | 310.6$/500mL (J&K) | 1.19 |
| Pd(PPh_3_)_4_ | 87mg | 36.4$/ 1 g (J&K) | 3.17 |
| NBS | 708.7mg | 95.3$/ 1kg (J&K) | 0.07 |
| Total Cost |  |  | 23.38 |
|  |  |  |  |
| Polymerization (1g) | | | |
| Reagent | Quantity | Unit(source) (By November 2020) | Cost ($) |
| Pd(PPh_3_)_4_ | 58mg | 36.4$/ 1 g (J&K) | 2.11 |
| **Overall cost for 1g PTVT-T** |  |  | **35.0** |

^a^Here, the reactants shown in Fig. S1 are taken into account, excluding organic solvents, inorganic substances (acids, bases and salt), solvents for post-treatment, water, electricity, rent and labor costs.

^b^The prices were obtained from some famous vendors, including J&k, Alfa Aesar, Sigma-Aldrich and TCI.

^c^The maximum package scales of few raw materials, including 5-(bromomethyl)undecane, 4,5-diaminophthalonitrile, tributyl(ethynyl)stannane and 2-bromothiophene-3-carboxylic acid, are limited to 25 g or 5 g. Thus, their unit are determined based on the maximum package scale.


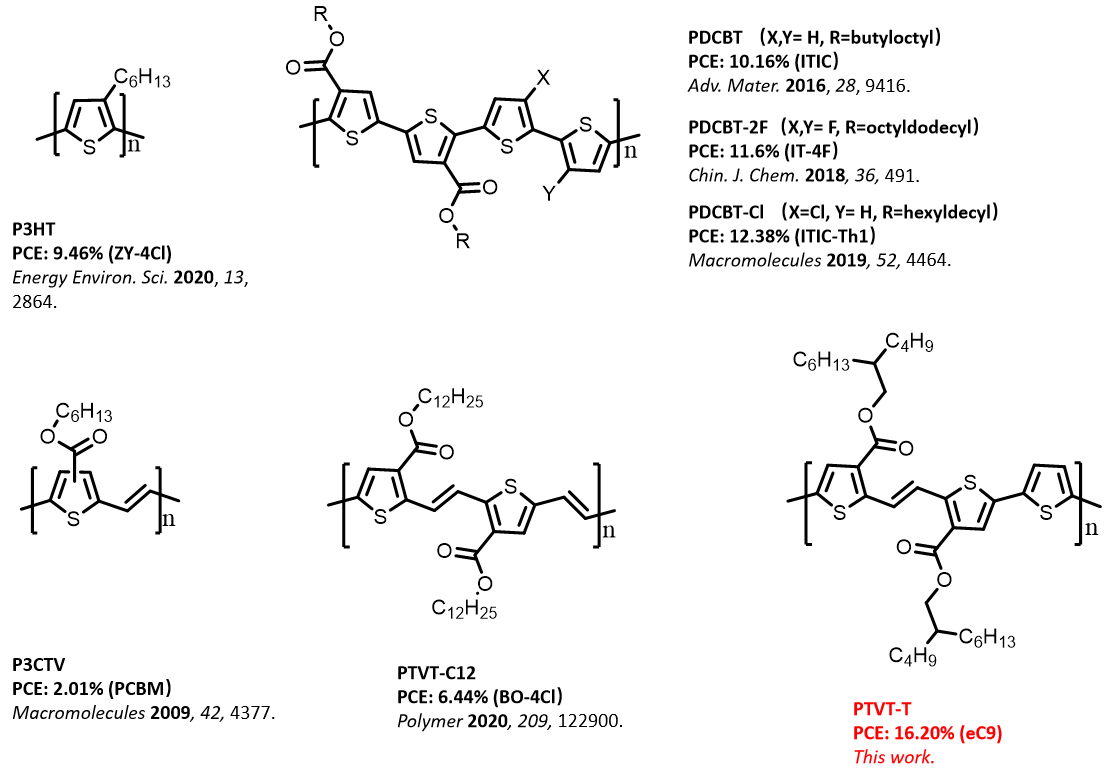


**Figure S2.** Molecular structures and corresponding photovoltaic performance of representative PT and PTV polymers and PTVT-T used in this work.


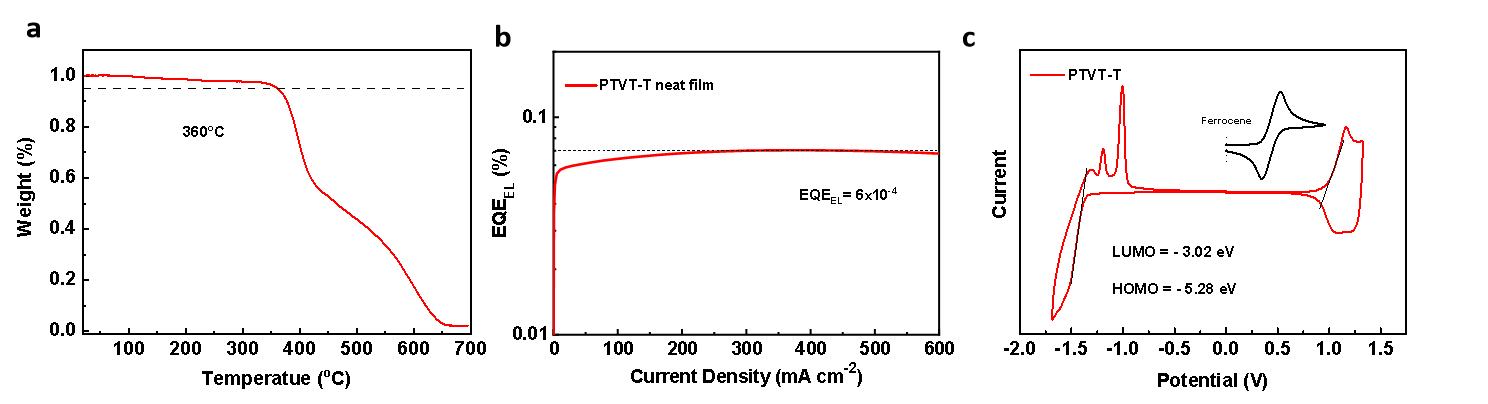


**Figure S3.** a) TGA curve under the air; b) EQE_EL_ curve; c) CV profile of PTVT-T.

**
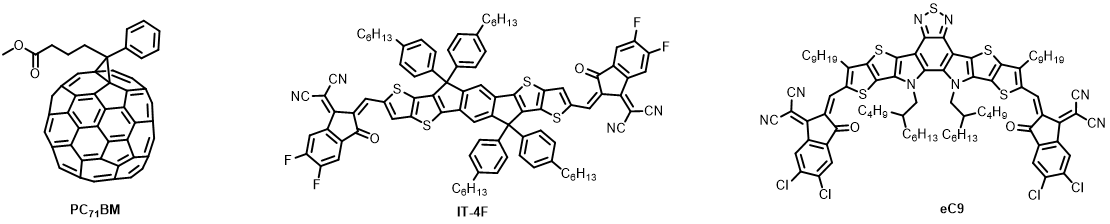
**

**Figure S4.** Chemical structures of PC_71_BM, IT-4F and eC9.

**
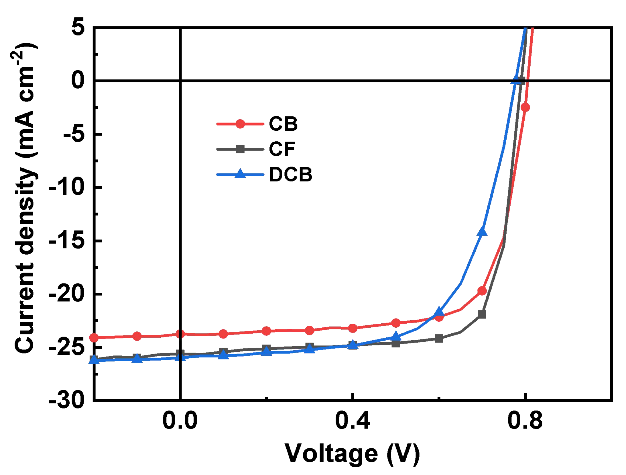
**

**Figure S5.** *J-V* curves of PTVT-T:eC9 based devices with different solvents.

**Table S2.** Optimization of solvents for PTVT-T:eC9 based devices.

| **Solvent** | ***V*_OC_ (V)** | ***J*_SC_ (mA cm^-2^)** | **FF** | **PCE_max_ (%)** |
| --- | --- | --- | --- | --- |
| CB | 0.79 | 25.62 | 0.76 | 15.34 |
| CF | 0.81 | 23.74 | 0.73 | 13.94 |
| *o*-DCB | 0.78 | 25.98 | 0.65 | 13.03 |

**
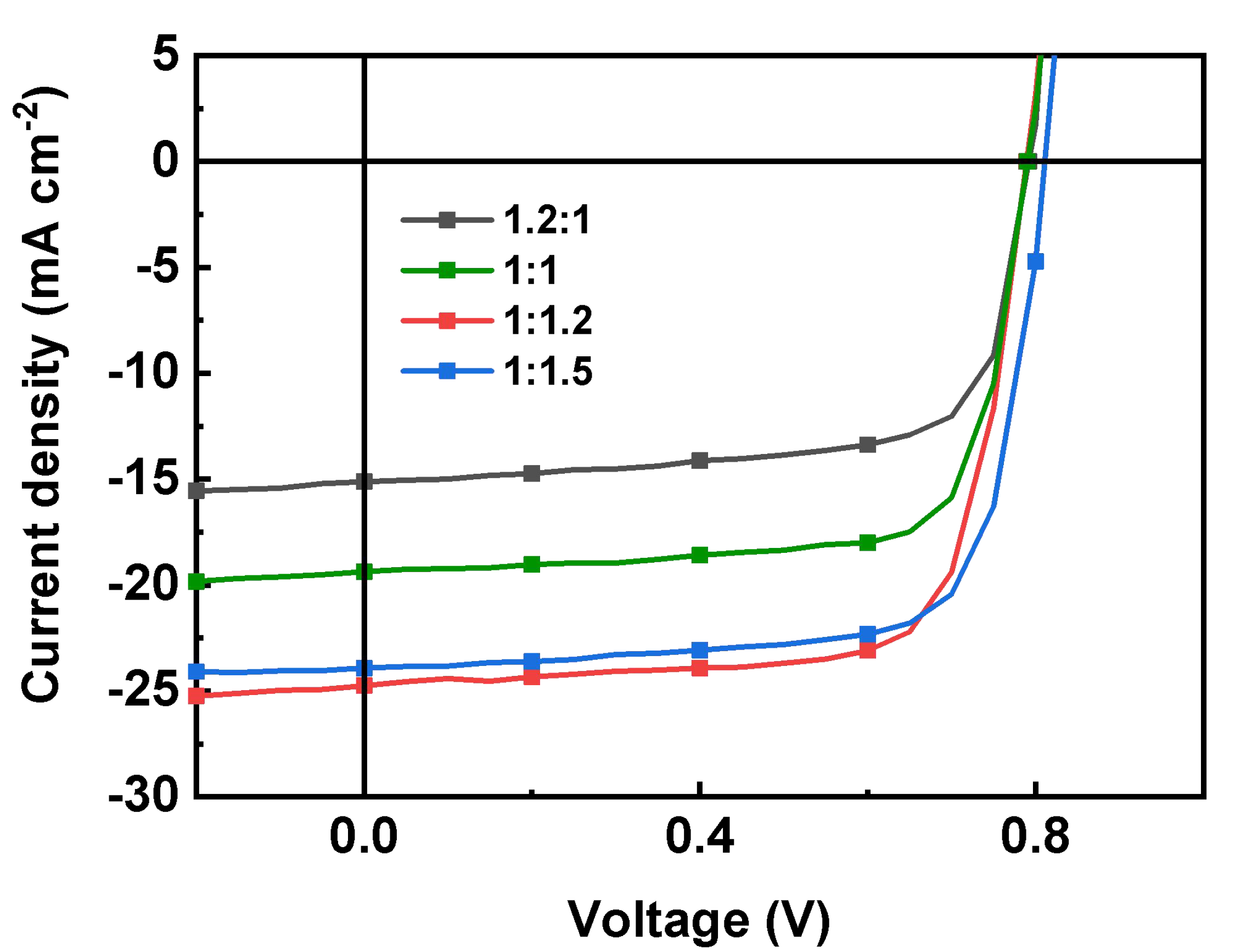
**

**Figure S6.** *J-V* curves of PTVT-T:eC9 based devices with different D/A ratios.

**Table S3.** Optimization of D/A ratios for PTVT-T:eC9 based devices.

| **D/A Ratios (w/w)** | ***V*_OC_ (V)** | ***J*_SC_ (mA cm^-2^)** | **FF** | **PCE_max_ (%)** |
| --- | --- | --- | --- | --- |
| 1.2:1 | 0.79 | 15.12 | 0.70 | 8.43 |
| 1:1 | 0.79 | 19.36 | 0.74 | 11.36 |
| 1:1.2 | 0.79 | 24.75 | 0.73 | 14.43 |
| 1:1.5 | 0.81 | 23.92 | 0.74 | 14.31 |


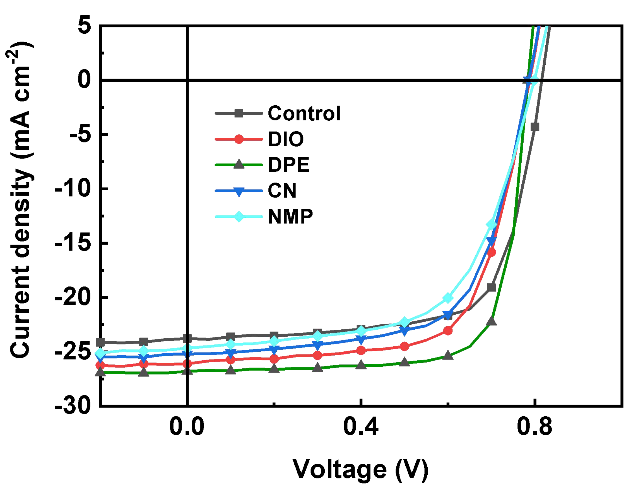


**Figure S7.** *J-V* curves of PTVT-T:eC9 based devices with different additives.

**Table S4.** Optimization of additives for PTVT-T:eC9 based devices.

| **Additives** | ***V*_OC_ (V)** | ***J*_SC_ (mA cm^-2^)** | **FF** | **PCE_max_ (%)** |
| --- | --- | --- | --- | --- |
| Control | 0.82 | 23.77 | 0.71 | 13.70 |
| DIO | 0.79 | 26.10 | 0.67 | 13.82 |
| DPE | 0.79 | 26.77 | 0.75 | 15.89 |
| CN | 0.79 | 25.20 | 0.65 | 12.92 |
| NMP | 0.80 | 24.66 | 0.61 | 12.02 |


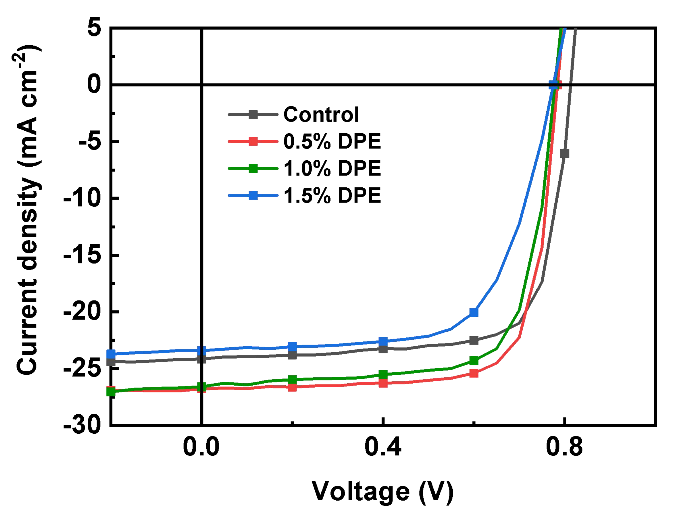


**Figure S8.** *J-V* curves of PTVT-T:eC9 based devices with different additive ratios.

**Table S5.** Optimization of additive ratios for PTVT-T:eC9 based devices.

| **Additive Ratios** | ***V*_OC_ (V)** | ***J*_SC_ (mA cm^-2^)** | **FF** | **PCE_max_（%）** |
| --- | --- | --- | --- | --- |
| Control | 0.81 | 24.15 | 0.75 | 14.70 |
| 0.5% DPE | 0.79 | 26.77 | 0.75 | 15.89 |
| 1% DPE | 0.78 | 26.59 | 0.73 | 15.10 |
| 1.5% DPE | 0.77 | 23.41 | 0.66 | 12.02 |


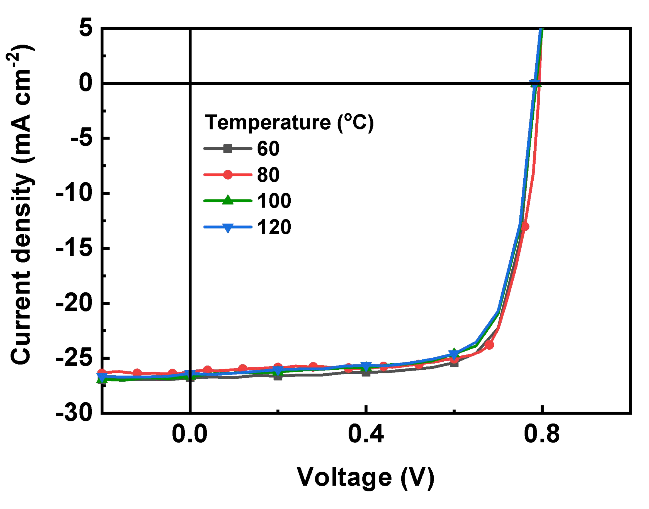


**Figure S9.** *J-V* curves of PTVT-T:eC9 based devices with different thermal annealing temperatures.

**Table S6.** Optimization of thermal annealing temperatures for PTVT-T:eC9 based devices.

| **TA Temperature (^o^C)** | ***V*_OC_ (V)** | ***J*_SC_ (mA cm^-2^)** | **FF** | **PCE_max_（%）** |
| --- | --- | --- | --- | --- |
| 60 | 0.79 | 26.48 | 0.72 | 15.25 |
| 80 | 0.79 | 26.22 | 0.78 | 16.20 |
| 100 | 0.79 | 26.77 | 0.75 | 15.89 |
| 120 | 0.78 | 26.37 | 0.74 | 15.28 |


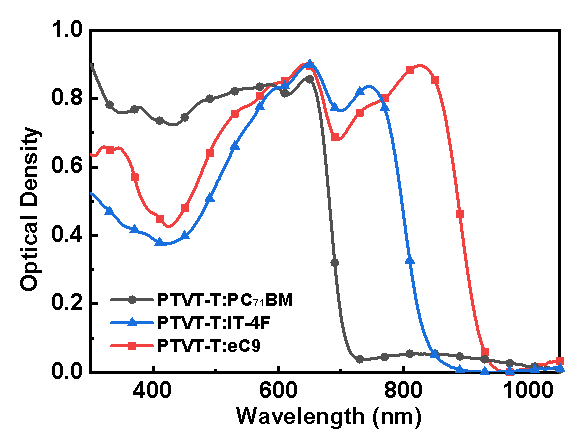


**Figure S10.** The absorption profile of PTVT-T:PC_71_BM, PTVT-T:IT-4F and PTVT-T:eC9 blend films.

**
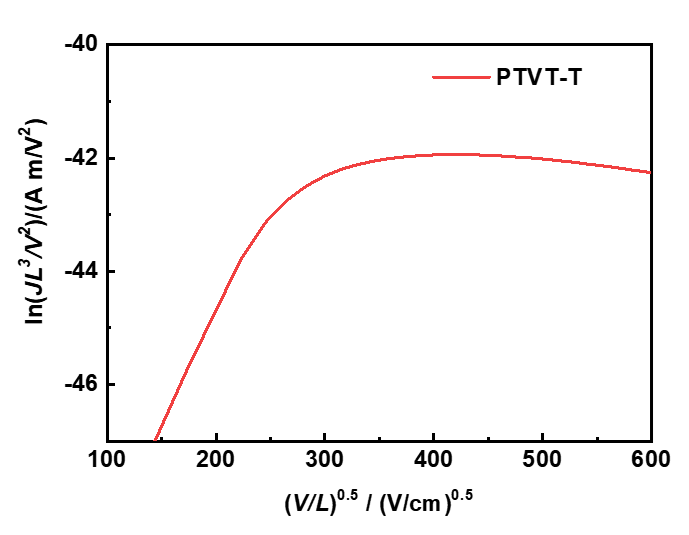
**

**Figure S11.** The dark *J-V* plots of the hole-only device based on PTVT-T.

**Table S7.** The hole, electron and Photo-CELIV mobilities of PTVT-T:PC_71_BM, PTVT-T:IT-4F and PTVT-T:eC9 based devices.

| **Active layer** | **Hole mobility**  ***μ*_h_ (cm^2^ V^−1^ s^−1^)** | **Electron mobility**  ***μ*_e_ (cm^2^ V^−1^ s^−1^)** | **Photo-CELIV mobility**  **(cm^2^ V^−1^ s^−1^)** |
| --- | --- | --- | --- |
| PTVT-T:PC_71_BM | 4.57 × 10^-4^ | 5.78 × 10^-4^ | 1.35 × 10^-4^ |
| PTVT-T:IT-4F | 1.83 × 10^-4^ | 1.39 × 10^-4^ | 5.32 × 10^-5^ |
| PTVT-T:eC9 | 2.46 × 10^-4^ | 2.31 × 10^-4^ | 1.23 × 10^-4^ |


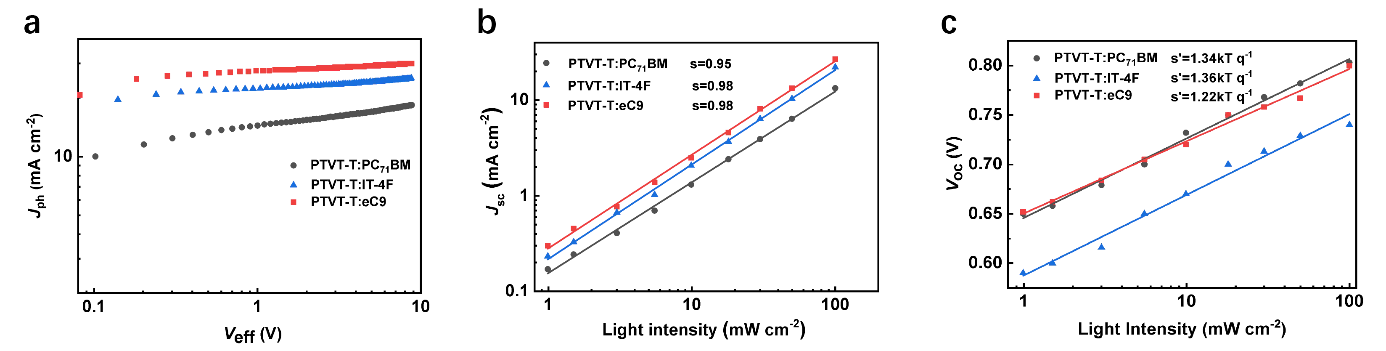


**Figure S12.** a) *J*_ph_ versus *V*_eff_ characteristics; *V*_OC_ (b) and *J*_SC_ (c) dependence on *P*_light_ of PTVT-T:PC_71_BM, PTVT-T:IT-4F and PTVT-T:eC9 based devices.


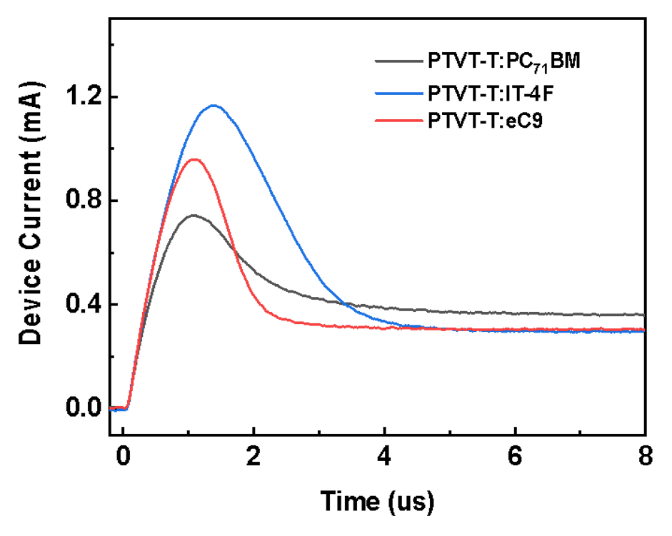


**Figure S13.** The photo-CELIV mobilities of PTVT-T:PC_71_BM, PTVT-T:IT-4F and PTVT-T:eC9 based devices.

**Table S8.** The GIWAXS parameters of PTVT-T neat film, PTVT-T:PC_71_BM, PTVT-T:IT-4F and PTVT-T:eC9 blends.

| **Films** | **100**  **(Å^-1^)** | ***d*-Spacing**  **(Å)** | **010**  **(Å^-1^)** | **FWHM**  **(Å^-1^)** | **CL**  **(Å)** |
| --- | --- | --- | --- | --- | --- |
| PTVT-T | 0.28 | 22.43 | 1.75 | 0.225 | 27.92 |
| PTVT-T:PC_71_BM | 0.29 | 21.66 | 1.75 | 0.242 | 25.95 |
| PTVT-T:IT4F | 0.29 | 21.66 | 1.75 | 0.160 | 39.24 |
| PTVT-T:eC9 | 0.30 | 20.93 | 1.75 | 0.233 | 26.95 |


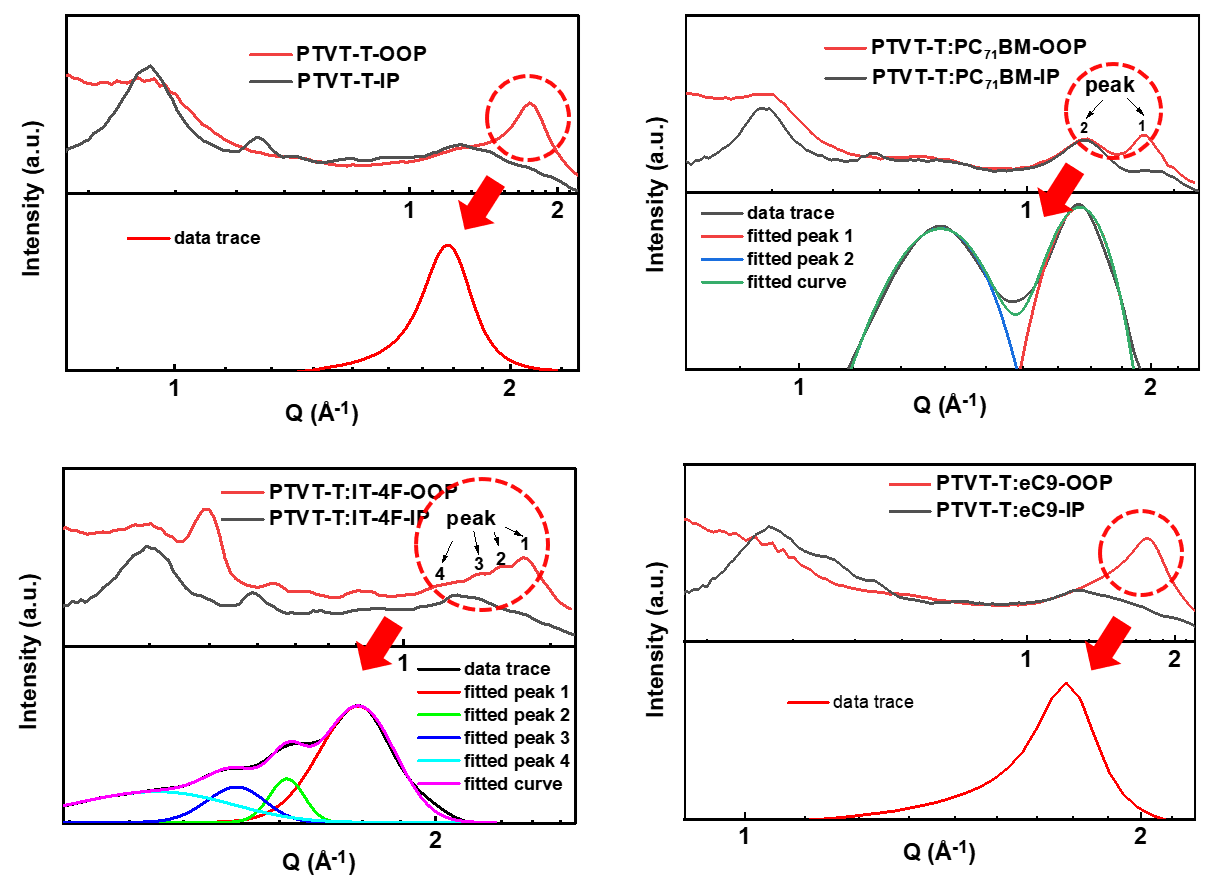


**Figure S14.** The fitting curves of GIWAXS.

**Table S9.** Detailed *E*_loss_ parameters of PTVT-T:PC_71_BM, PTVT-T:IT-4F and PTVT-T:eC9 based OSCs.

| **Devices** | $\boldsymbol{E}_{\boldsymbol{g}}$ **(eV)** | $\boldsymbol{E}_{\boldsymbol{loss}}$ **(eV)** | $\boldsymbol{qV}_{\boldsymbol{OC}}^{\boldsymbol{rad}}$ **(eV)** | $\boldsymbol{\Delta}\boldsymbol{E}_{\boldsymbol{1}}$ **(eV)** | $\boldsymbol{\Delta}\boldsymbol{E}_{\boldsymbol{2}}$ **(eV)** | $\boldsymbol{\Delta}\boldsymbol{E}_{\boldsymbol{3}}$ **(eV)** |
| --- | --- | --- | --- | --- | --- | --- |
| PTVT-T:PC_71_BM | 1.79 | 0.950 | 1.502 | 0.288 | 0.328 | 0.334 |
| PTVT-T:IT-4F | 1.51 | 0.760 | 1.240 | 0.270 | 0.180 | 0.310 |
| PTVT-T:eC9 | 1.40 | 0.610 | 1.137 | 0.263 | 0.106 | 0.241 |


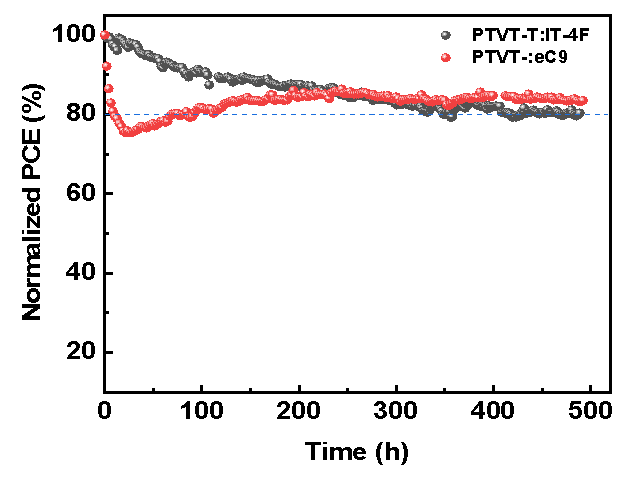


**Figure S15**. The stability of the devices based on PTVT-T:IT-4F and PTVT-T:eC9 under continues AM 1.5G illumination.

**Reference**

1. M. J. Frisch, GWT, H. B. Schlegel, G. E. Scuseria, M. A. Robb, J. R. Cheeseman, G. Scalmani, V. Barone, B. Mennucci, G. A. Petersson, H. Nakatsuji, M. Caricato, X. Li, H. P. Hratchian, A. F. Izmaylov, J. Bloino, G. Zheng, J. L. Sonnenberg, M. Hada, M. Ehara, K. Toyota, R. Fukuda, J. Hasegawa, M. Ishida, T. Nakajima, Y. Honda, O. Kitao, H. Nakai, T. Vreven, J. A. Montgomery Jr., J. E. Peralta, F. Ogliaro, M. J. Bearpark, J. Heyd, E. N. Brothers, K. N. Kudin, V. N. Staroverov, R. Kobayashi, J. Normand, K. Raghavachari, A. P. Rendell, J. C. Burant, S. S. Iyengar, J. Tomasi, M. Cossi, N. Rega, N. J. Millam, M. Klene, J. E. Knox, J. B. Cross, V. Bakken, C. Adamo, J. Jaramillo, R. Gomperts, R. E. Stratmann, O. Yazyev, A. J. Austin, R. Cammi, C. Pomelli, J. W. Ochterski, R. L. Martin, K. Morokuma, V. G. Zakrzewski, G. A. Voth, P. Salvador, J. J. Dannenberg, S. Dapprich, A. D. Daniels, Ö. Farkas, J. B. Foresman, J. V. Ortiz, J. Cioslowski, D. J. Fox. *Gaussian, Inc, Wallingford, CT, USA,*. 2009.

2. Liu, J, Chen, S, Qian, D*, et al.* Fast charge separation in a non-fullerene organic solar cell with a small driving force. *Nat Energy*. 2016; **1**: 16089.

3. Xu, S, Wang, X, Feng, L*, et al.* Optimizing the conjugated side chains of quinoxaline based polymers for nonfullerene solar cells with 10.5% efficiency. *J Mater Chem A*. 2018; **6**: 3074-83.

4. Zhang, S, Qin, Y, Zhu, J*, et al.* Over 14% Efficiency in Polymer Solar Cells Enabled by a Chlorinated Polymer Donor. *Adv Mater*. 2018; **30**: 1800868.

5. Zhang, M, Guo, X, Zhang, S*, et al.* Synergistic effect of fluorination on molecular energy level modulation in highly efficient photovoltaic polymers. *Adv Mater*. 2014; **26**: 1118-23.

6. Zong, K, Kim, H. A Convenient Synthesis of Benzo[1,2-b:6,5-b′:3,4-c′′]trithiophenes Starting from Thiophene. *Synthesis*. 2018; **51**: 859-64.

7. Cui, C, Fan, X, Zhang, M*, et al.* A D-A copolymer of dithienosilole and a new acceptor unit of naphtho[2,3-c]thiophene-4,9-dione for efficient polymer solar cells. *Chem Commun*. 2011; **47**: 11345-7.

8. Qian, D, Ye, L, Zhang, M*, et al.* Design, Application, and Morphology Study of a New Photovoltaic Polymer with Strong Aggregation in Solution State. *Macromolecules*. 2012; **45**: 9611-7.

9. Han, YW, Jeon, SJ, Lee, HS*, et al.* Evaporation‐Free Nonfullerene Flexible Organic Solar Cell Modules Manufactured by An All‐Solution Process. *Adv Energy Mater*. 2019; **9**: 1902065.

10. Zhang, M, Guo, X, Ma, W*, et al.* A Large-Bandgap Conjugated Polymer for Versatile Photovoltaic Applications with High Performance. *Adv Mater*. 2015; **27**: 4655-60.

11. Arroyave, FA, Richard, CA, Reynolds, JR. Efficient Synthesis of Benzo[1,2-b:6,5-b′]dithiophene-4,5-dione (BDTD) and Its Chemical Transformations into Precursors for π-Conjugated Materials. *Org Lett*. 2012; **14**: 6138-41.

12. Mei, C-Y, Liang, L, Zhao, F-G*, et al.* A Family of Donor–Acceptor Photovoltaic Polymers with Fused 4,7-Dithienyl-2,1,3-benzothiadiazole Units: Effect of Structural Fusion and Side Chains. *Macromolecules*. 2013; **46**: 7920-31.

13. Lee, J, Sin, DH, Clement, JA*, et al.* Medium-Bandgap Conjugated Polymers Containing Fused Dithienobenzochalcogenadiazoles: Chalcogen Atom Effects on Organic Photovoltaics. *Macromolecules*. 2016; **49**: 9358-70.

14. Liu, Q, Jiang, Y, Jin, K*, et al.* 18% Efficiency organic solar cells. *Sci Bull*. 2020; **65**: 272-5.

15. Yan, H, Zhao, J. C(SP3)-C(SP2) CROSS-COUPLING REACTION OF ORGANOZINC REAGENTS AND HETEROCYCLIC (PSEUDO)HALIDES[P]. *WO2018019291 (A1), 2018-08-02*.

16. Liu, Y, Li, X, Zhu, W*, et al.* A2-(pi-A1)2 wide band gap non-fullerene receptor material based on diphenylthiophenyl sulfone and preparation method and application thereof *CN109081825 (A),2018-12-25*.

17. Sun, C, Pan, F, Bin, H*, et al.* A low cost and high performance polymer donor material for polymer solar cells. *Nat Commun*. 2018; **9**: 743.

18. Sun, C, Qin, S, Wang, R*, et al.* High Efficiency Polymer Solar Cells with Efficient Hole Transfer at Zero Highest Occupied Molecular Orbital Offset between Methylated Polymer Donor and Brominated Acceptor. *J Am Chem Soc*. 2020; **142**: 1465-74.

19. Ozdemir, M, Choi, D, Kwon, G*, et al.* Solution-Processable BODIPY-Based Small Molecules for Semiconducting Microfibers in Organic Thin-Film Transistors. *ACS Appl Mater Interfaces*. 2016; **8**: 14077-87.

20. Pomerantz, M, Turkman, N. A Facile and Improved Synthesis of 3-Fluorothiophene. *Synthesis*. 2008; **2008**: 2333-6.

21. Yu, J, Ornelas, JL, Tang, Y*, et al.* 2,1,3-Benzothiadiazole-5,6-dicarboxylicimide-Based Polymer Semiconductors for Organic Thin-Film Transistors and Polymer Solar Cells. *ACS Appl Mater Interfaces*. 2017; **9**: 42167-78.

22. Fan, B, Li, M, Zhang, D*, et al.* Tailoring Regioisomeric Structures of π-Conjugated Polymers Containing Monofluorinated π-Bridges for Highly Efficient Polymer Solar Cells. *ACS Energy Lett*. 2020; **5**: 2087-94.

23. Feng, L-W, Chen, J, Mukherjee, S*, et al.* Readily Accessible Benzo[d]thiazole Polymers for Nonfullerene Solar Cells with >16% Efficiency and Potential Pitfalls. *ACS Energy Lett*. 2020; **5**: 1780-7.

24. Zhou, H, Yang, L, Stuart, AC*, et al.* Development of fluorinated benzothiadiazole as a structural unit for a polymer solar cell of 7 % efficiency. *Angewandte Chemie*. 2011; **50**: 2995-8.

25. Yu, C-Y, Hsu, C-C, Weng, H-C. Synthesis, characterization, aggregation-induced emission, solvatochromism and mechanochromism of fluorinated benzothiadiazole bonded to tetraphenylethenes. *RSC Advances*. 2018; **8**: 12619-27.

26. Hu, H, Jiang, K, Yang, G*, et al.* Terthiophene-based D-A polymer with an asymmetric arrangement of alkyl chains that enables efficient polymer solar cells. *J Am Chem Soc*. 2015; **137**: 14149-57.
